# Fermented plant-based yogurt alternatives in Switzerland: nutritional adequacy and fortification practices

**DOI:** 10.3389/fnut.2026.1899857

**Published:** 2026-08-11

**Authors:** Nicole Barth, Leonie H. Bogl, Isabelle Herter-Aeberli

**Affiliations:** 1 Laboratory of Nutrition and Metabolic Epigenetics, Department of Health Sciences and Technology, Institute of Food, Nutrition and Health, ETH Zurich, Zurich, Switzerland; 2 Division of Nutrition and Dietetics, Department of Health Professions, Bern University of Applied Sciences, Bern, Switzerland

**Keywords:** dairy, dietary substitution, market survey, micronutrient fortification, plant-based alternatives, Switzerland, vegan, yogurt

## Abstract

**Background:**

Global dietary guidelines increasingly advocate a shift toward plant-based eating patterns, paralleled by growing consumer demand for plant-based dairy alternatives. This study characterized the nutritional composition of fermented plant-based yogurt alternatives on the Swiss market and assessed the micronutrient implications of replacing dairy yogurt with these products.

**Methods:**

A cross-sectional market survey of fermented plant-based yogurt and yogurt-like products was conducted across major Swiss retailers in 2025. Nutrient composition and micronutrient fortification were extracted from product labels. Substitution modeling using micronutrient data from the Swiss Food Composition Database, Swiss national consumption data, and national dietary reference values was applied to compare dairy yogurt with soy- and coconut-based alternatives.

**Results:**

A total of 98 products were identified, dominated by soy- 36.6 % and coconut-based 26.8 % formulations. Soy-based products most closely approximated dairy yogurt in protein content while providing lower total energy and saturated fat, whereas coconut-based alternatives were more energy-dense, higher in saturated fat, and lower in protein. Only 18.4 % of products were fortified with micronutrients. Substitution modeling showed that 1:1 replacement of dairy yogurt with unfortified plant-based alternatives reduced intakes of calcium, iodine, phosphorus, zinc, vitamins A, B_2_, B_5_, and B_12_, while folate intake increased when soy-based alternatives were used.

**Conclusion:**

Fermented plant-based yogurt alternatives are widely available in Switzerland but vary substantially in nutritional composition, and limited fortification means these products are not nutritionally interchangeable with dairy yogurt. Achieving a nutritionally adequate shift toward plant-based alternatives will require improved fortification practices and evidence-based consumer guidance.

## 1 Introduction

Global food systems face increasing pressure to deliver adequate nutrition while remaining within planetary boundaries, as food production is projected to substantially increase greenhouse gas emissions and land-use demands under current dietary trends (1). These considerations underpin calls for a “Great Food Transformation” aligned with the UN Sustainable Development Goals and the Paris Agreement (2). Building on this premise, the updated EAT-Lancet Commission emphasizes the Planetary Health Diet (PHD) as a framework for achieving both environmental sustainability and human health. The PHD prioritizes plant foods, complemented by modest amounts of fish, dairy, and meat. Although current dietary patterns diverge substantially from this model, widespread adoption of the PHD has been projected to prevent up to 15 million premature deaths annually (3).

Despite the growing emphasis on plant-based diets, fermented dairy products such as yogurt remain nutritionally relevant due to their well-documented health benefits. Fermentation generates bioactive metabolites and delivers live microorganisms with probiotic potential, leading to more consistent positive health associations than those observed for non-fermented dairy. Regular consumption of fermented milks has been associated with reduced risks of several chronic diseases, including type 2 diabetes and colorectal cancer, as well as benefits for cardiovascular, bone, and gastrointestinal health, reflecting fermentation-related improvements in nutrient bioavailability and microbial functionality (4, 5). Yet these health benefits must be weighed against the substantial environmental footprint of dairy production. A robust body of evidence shows that plant-based dairy alternatives, such as soy milk, have markedly lower environmental impacts than animal-source products across multiple indicators, including greenhouse gas emissions and land use (6). Consequently, substituting dairy with plant-based alternatives is increasingly recognized as an accessible strategy to reduce the environmental footprint of diets (7). This tension between nutritional benefits and environmental impacts has driven growing interest in fermented plant-based yogurt alternatives designed to replicate the functional and sensory properties of dairy yogurt. While dairy yogurt is inherently a fermented product, plant-based yogurt alternatives vary in their production methods, though the available evidence suggests that commercially available products are almost universally fermented (8). By fermenting diverse plant substrates, these products aim to deliver probiotics and fermentation-derived bioactives while achieving yogurt-like texture and palatability, supporting their rapid emergence as functional foods in the retail market (9).

Switzerland offers an interesting perspective on the tensions between a strong dairy tradition and the emergence of plant-based dietary trends. Average dairy yogurt consumption is approximately 75 g/day, with national recommendations of 175 g/day, substantially exceeding the global mean of 20 g/day (10–12). At the same time, dietary patterns are shifting, as the proportion of flexitarians and ovolacto-vegetarians is increasing (13). Correspondingly, the Swiss market for dairy alternatives has expanded rapidly, ranking third in Europe for per-capita spending on dairy substitute beverages by 2021, and yogurt alternatives accounting for 6 % of total yogurt sales (14).

Fermented plant-based yogurt alternatives present challenges and opportunities distinct from those of dairy yogurt. As a result of the wide range of substrates used for their production, such as cereals, pseudocereals, legumes, nuts, and grains, the raw materials for plant-based yogurt alternative formulations differ substantially from milk, resulting in highly variable nutritional compositions (9). While plant-based yogurt alternatives to dairy clearly present a way toward improving dietary sustainability, uncertainties remain regarding the nutritional adequacy of fermented plant-based yogurt alternatives when used as substitutes for dairy yogurt.

To address these gaps, we conducted a systematic assessment of fermented plant-based yogurt alternatives in Switzerland. Specifically, our study aimed to:

(i) characterize the availability of fermented plant-based yogurt and yogurt-like products on the Swiss market,
(ii) analyze their nutrient composition and micronutrient fortification based on product labeling, and
(iii) model the potential impact of replacing dairy yogurt with these products on micronutrient intake, using nationally representative consumption patterns and national dietary recommendations.

Together, these analyses offer a timely assessment of whether current fermented plant-based yogurt alternatives in Switzerland can adequately support the nutritional needs associated with a shift toward more sustainable, predominantly plant-based diets.

## 2 Materials and methods

### 2.1 Market survey on fermented plant-based yogurt alternatives in the Swiss market

#### 2.1.1 Sampling and search method

We conducted a market analysis focusing on plant-based fermented yogurt and yogurt-like products containing viable microorganisms, chosen based on their capacity of gastrointestinal survival, transient colonization and potential health effects (15, 16). To achieve this, a cross-sectional market survey was completed over a defined 3-month period (September-November 2025) to minimize temporal fluctuations in product availability. All products meeting the predefined inclusion criteria were systematically cataloged using standardized templates and harmonized extraction procedures.

#### 2.1.2 Operational definition of products containing viable microorganisms

A product was classified as “containing viable microorganisms” if at least one of the following criteria was met: (I) Explicit label statements indicating microbial viability (e.g., “unpasteurized,” “raw,” “with live cultures,” “naturally fermented”) together with declared cold-chain requirements, (II) Starter cultures listed in the ingredient list, (III) Manufacturer claims confirming the presence of viable microorganisms at point of sale. Products subjected to post-fermentation heat treatment (e.g., pasteurization, sterilization, heat stabilization) were excluded. Viability was not independently verified through laboratory testing.

#### 2.1.3 Retailer selection and search strategy

To ensure broad market coverage, three retail strata were systematically screened. The survey was conducted in national full-line grocers and discounters: Migros, Coop, Denner, Aldi, Lidl, Spar, Volg; nation-wide online retailers and vegan specialty online shops: Galaxus.ch, Farmy.ch, MrVegan.ch, Fabulous.ch, HelloVegan.ch and organic and specialty food stores (primarily in Zurich): Alnatura: Alnatura branches at Zürich HB Sihlquai, Zürich Limmatplatz, Zürich Höngg; Vitus Biomarkthalle, L'Ultimo Bacio, BachserMärt Seefeld, Reformhaus Vier Linden, BRIDGE. These outlets collectively reflect the Swiss retail landscape, where Coop and Migros account for the largest market shares (35.1 % and 34.8 %), with Denner, Volg and Galaxus/Digitec contributing an additional 9.1 %, 4.2 % and 4 %, respectively (17).

A combined online and in-store strategy was used. During an initial online screening, retailer websites and apps were screened using category navigation and keyword searches (“vegan,” “plant-based,” “yogurt alternative,” “soy,” “almond,” “cashew,” “coconut,” “oat”) in German and English to reflect common labeling practices. All retrieved products were assessed against the predefined operational criteria. Second, in-store audits were performed, where **one** to three major outlets of each chain were visited to confirm product availability, shelf placement, cold-chain status, and label information.

#### 2.1.4 Inclusion and exclusion criteria

Products were included if they were (i) plant-based fermented foods containing viable microorganisms and (ii) marketed as yogurt alternatives or yogurt-like products. Items labeled as “Quark,” “Kefir,” “Full-fat quark,” “Skyr,” or “Greek-style,” or related terminology were classified as “yogurt-like alternatives.” We report yogurt alternatives and yogurt-like alternatives separately for macronutrients and fortification; yogurt-like products are included to reflect consumer-facing substitution patterns but are not treated as nutritionally equivalent. Both organic and conventional products were eligible and included regardless of their availability across retail types.

Products were excluded if they were non-fermented, subjected to post-fermentation heat treatment (e.g., pasteurization or sterilization), non-vegan, or classified as dietary supplements or microbial starter cultures. Items with identical formulations but offered in multiple package sizes were treated as a single product, all sizes were documented, but only the smallest or standard unit was used for analyses. Duplicate items occurring across different retailers were consolidated into a single entry.

#### 2.1.5 Data collection and variables

For each eligible product, standardized data about product characteristics, ingredients and nutritional information were extracted to ensure comparability across retailers. Product-level variables included the name and description, brand, pack size (g/mL), production location, storage conditions, plain vs. flavored classification, product URL (if available), and the date of documentation. Market variables comprised retailer availability across all screened outlets and price, recorded both as the unit price (CHF) and as a normalized price per 100 g or 100 mL. Ingredient-based information was captured using a structured classification scheme. The main substrate was assigned, while added plant protein powders were not considered substrates. Additives were coded into predefined categories, including antioxidants, thickeners/stabilizers/emulsifiers and acidity regulators. Fortification compounds were captured when declared. Additional declared ingredients were documented, such as flavorings or coloring agents. Nutritional information was extracted per 100 g and included energy, fat, saturated fats, carbohydrates, total sugars, fiber, protein, salt, calcium (with the chemical form when available), and vitamins D, B_12_, and B_2_. Labels report only total sugars, so added sugars were not quantified but identified qualitatively from the ingredient list. Values reported as thresholds (e.g., “ < 0.5”) were recorded as half of the stated value.

### 2.2 Nutrient content and content of fortificants

In this study, nutritional adequacy refers to the extent to which fermented plant-based yogurt alternatives approximate the macro- and micronutrient contributions of dairy yogurt when evaluated using label-derived composition data and Swiss dietary reference values. Here, the median macronutrient content was calculated separately for plain yogurt alternatives and yogurt-like alternatives, with additional stratification by substrate for yogurt alternatives. For products containing added micronutrients, the concentration of fortified nutrients was derived from the values declared on the nutrition labels and summarized across all fortified products. For comparison to dairy yogurt, nutrient values for conventional dairy yogurt were obtained from the Swiss Food Composition Database (18) for a full-fat cow's-milk yogurt.

### 2.3 Micronutrient substitution modeling

To estimate the impact of replacing dairy yogurt with plant-based alternatives on micronutrient intake, nutrient substitution was modeled using analytical composition data from the Swiss Food Composition Database. Modeling was restricted to micronutrients, as macronutrient data were available from product labels and addressed descriptively, whereas micronutrient composition required database-derived values and dedicated modeling to assess potential intake gaps. Micronutrient values for dairy yogurt and for coconut- and soy-based yogurt alternatives were extracted from the database (18), and the median values used in the modeling are presented in Table 1.

**Table 1:** Micronutrient content (per 100 g) of plain dairy yogurt and fermented plain soy- and coconut-based yogurt alternatives, shown alongside the recommended dietary allowance (RDA) values for adults based on the recommendations of the Federal Food Safety and Veterinary Office, minimum and maximum ranges were reported to reflect sex-specific intake requirements (20).

Micronutrient	RDA (adults)	Unit	Micronutrient content per 100 g		
			Dairy yogurt, plain, full-fat	Soy yogurt alternative, plain	Coconut yogurt alternative, plain
Vitamin A activity, RE	650–750	μg	39	1	1
Vitamin B_1_ (Thiamine)	0.84	mg	0.02	0.01	0
Vitamin B_2_ (Riboflavin)	1.6	mg	0.18	0.005	0
Vitamin B_3_ (Niacin)	13	mg	0.09	0.05	0.1
Vitamin B_5_ (Pantothenic acid)	5	mg	0.33	0.04	0.03
Vitamin B_6_ (Pyridoxine)	1.6–1.7	mg	0.04	0.005	0
Vitamin B_9_ (Folate)	330	μg	5	18.6	0.5
Vitamin B_12_ (Cobalamin)	4	μg	0.3	0	0
Vitamin C (Ascorbic acid)	95–110	mg	2	0.25	0
Vitamin D (Calciferol)	15	μg	0.1	0.15	0
Vitamin E (α-Tocopherol)	11–13	mg	0.1	0.23	0
Potassium (K)	3,500	mg	170	110	92
Sodium (Na)	2,000	mg	49	34	25
Chloride (Cl)	3,100	mg	120	24	68
Calcium (Ca)	950–1,000	mg	140	14	5
Magnesium (Mg)	300–350	mg	12	17	9.7
Phosphorus (P)	550	mg	110	48	22
Iron (Fe)	11–16	mg	0	0.4	0.15
Iodine (I)	150	μg	16	n.d.	n.d.
Zinc (Zn)	7.5–16.3	mg	0.4	0.3	0.1

Median micronutrient values were obtained from the Swiss Food Composition Database (18).

#### 2.3.1 Baseline dietary values and modeling assumptions

Baseline micronutrient intake was estimated using yogurt consumption data from menuCH (10), representing current dietary patterns in Switzerland. Recommended intake values were derived from national dietary guidelines as specified in the Swiss Food Pyramid (12). To ensure comparability and avoid confounding by added sugars, flavors, or other additives, the modeling was restricted to plain, unsweetened dairy and fermented plant-based yogurt alternatives.

The following micronutrients were assessed: vitamin A (RE), vitamin B_1_ (Thiamine), vitamin B_2_ (Riboflavin), vitamin B_3_ (Niacin), vitamin B_5_ (Pantothenic acid), vitamin B_6_ (Pyridoxine), vitamin B_9_ (Folate), vitamin B_12_ (Cobalamin), vitamin C (Ascorbic acid), vitamin D (Calciferol), vitamin E (α-Tocopherol), potassium (K), sodium (Na), chloride (Cl), calcium (Ca), magnesium (Mg), phosphorus (P), iron (Fe), iodine (I), zinc (Zn).

#### 2.3.2 Selection of yogurt types for substitution modeling

To capture the variability in plant-based yogurt formulations present on the Swiss market, the modeling strategy incorporated selected representative products corresponding to the main substrate groups identified in the market survey. Three representative yogurt types were selected. Plain whole-milk yogurt (3.5 % fat) from the Swiss Food Composition Database served as the dairy reference, reflecting its higher consumption relative to low-fat yogurts in Switzerland (19). Unfortified soy and coconut yogurt alternatives were modeled as representative non-fortified plant-based products, enabling evaluation of nutrient gaps associated with formulations lacking protein and micronutrient enrichment. This decision was based on our observation that only 18.4 % of fermented plant-based yogurt and yogurt-like alternatives available on the Swiss market were fortified, making fortification rather an exception than the norm. Modeling unfortified products therefore provides a conservative and market-representative estimate of micronutrient intake under typical consumer conditions.

Micronutrient intake was modeled under three scenarios: (1) the current status quo, reflecting omnivorous consumption in which all yogurt intake is dairy-based, (2) assuming a 1:1 substitution by weight with soy yogurt alternatives, and (3) assuming a 1:1 substitution by weight with coconut yogurt alternatives. The impact of substitution was evaluated by comparing the proportion of the recommended dietary allowance (RDA) supplied by each yogurt type for every micronutrient assessed. RDA values for adults of 18–65 years for Switzerland were obtained from published recommendations of the Federal Food Safety and Veterinary Office (FSVO). Minimum and maximum ranges were reported to reflect sex-specific intake requirements (20).

### 2.4 Statistical analyses

All statistical analyses were conducted using R statistical software (version 4.5.2). Nutrient data obtained from the product labels were first examined descriptively, reporting the median together with the minimum and maximum values for each substrate group. The distribution of each nutrient variable was assessed for normality using the Shapiro-Wilk test, supplemented by visual inspection of histograms and density plots stratified by substrate type. As data were not normally distributed and several groups had small sample sizes, non-parametric methods were used.

For comparisons between fermented plant-based yogurt alternatives and dairy yogurt, which served as the reference value, we applied a one-sample Wilcoxon Signed-Rank Test. This non-parametric test was used to evaluate whether nutrient values in each plant-based substrate differed significantly from the median value of the dairy yogurt group. Tests were performed separately for each nutrient and substrate type. Due to the small sample sizes across various groups, the exact *p*-values were calculated for the one-sample Wilcoxon Signed-Rank Test. Substrates with insufficient sample sizes to achieve statistical significance (e.g. *n* ≤ 5) were included for descriptive analysis only. Significance was set at *p* < 0.05. Given the number of nutrient-specific comparisons against a fixed dairy yogurt reference, *p*-values are interpreted as exploratory.

## 3 Results

### 3.1 Market structure and product characterization

We identified a total of 98 products which are presented in Supplementary Material S1, of which yogurt alternatives (total *n* = 82, *n* = 24 plain, *n* = 58 flavored) constituted the largest part, while yogurt-like alternatives (total *n* = 16, *n* = 8 plain, *n* = 8 flavored) were a minority. The main substrate for yogurt alternatives was soy (*n* = 30) which accounted for 36.6 % of the products, followed by coconut (*n* = 22, 26.8 %), mixed substrate (*n* = 15, 18.3 %), oat (*n* = 9, 11 %), and almond (*n* = 6, 7.3 %). For yogurt-like alternatives, soy was the main substrate (*n* = 15) and only one product was coconut based. In total, we identified 14 brands, all based in Europe. The location of production was similarly confined to Europe, with most products manufactured in Switzerland (44.9 %; *n* = 44), followed by France (17.3 %; *n* = 17), Germany (15.3 %; *n* = 15), Austria (12.2 %, *n* = 12), Belgium (9.2 %; *n* = 9) and Poland (1.0 %, *n* = 1).

The median normalized price of all yogurt alternatives and yogurt-like alternatives combined was 1.00 CHF per 100 g. The median normalized price of yogurt alternatives was 0.99 CHF per 100 g and the median normalized price of all yogurt-like alternatives was 1.14 CHF per 100 g. The normalized price for the “plain” options of all yogurt alternatives and yogurt-like alternatives combined was 0.86 CHF per 100 g, whereas the median normalized price for “flavored” products was 1.10 CHF per 100 g. The median price normalized per substrate for all yogurt alternatives and yogurt-like alternatives combined differed, with the most expensive being oat-based products (1.60 CHF per 100 g), followed by mixed-substrate (1.10 CHF per 100 g), almond (1.00 CHF per 100 g) and coconut (0.90 CHF per 100 g), while soy-based yogurt alternatives were available at the lowest prices of all substrates (0.78 CHF per 100 g).

### 3.2 Macronutrient composition within the nutritional characterization of products

**Figure 1:**
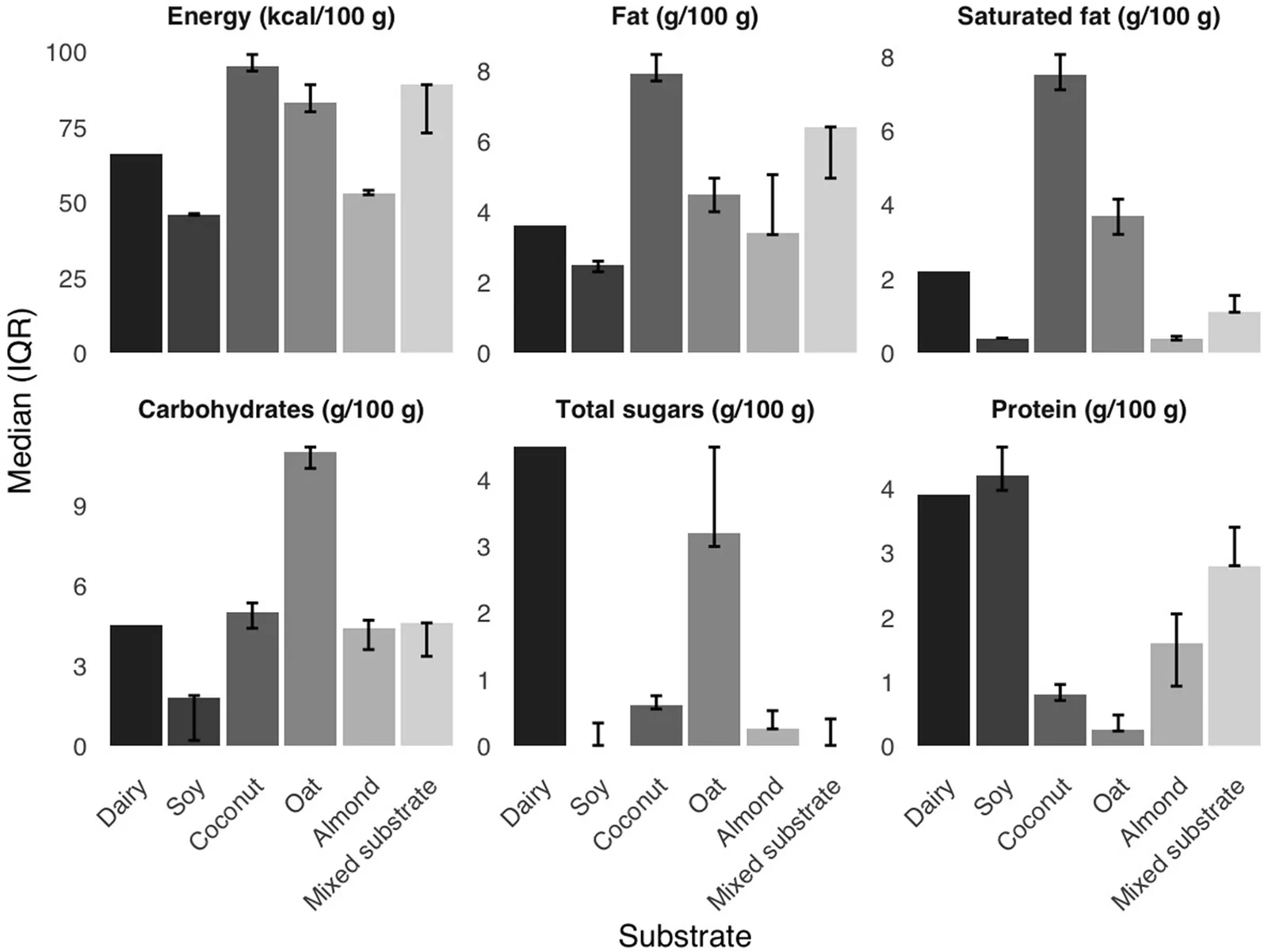
Median macronutrient content (per 100 g) of plain fermented plant-based yogurt alternatives by substrate, compared with dairy yogurt as the reference. Bars show medians and error bars the interquartile range (IQR), dairy is a single value from the Swiss Food Composition Database (18). Number of products per substrate: soy (*n* = 8), coconut (*n* = 7), oat (*n* = 3), almond (*n* = 3), mixed (*n* = 3).

Macronutrient content of plain yogurt alternatives across the five substrate categories (*n*=24) in comparison to dairy yogurt is shown in Figure 1. The corresponding descriptive data and salt content (median, full range and per-substrate sample size) together with the pairwise comparisons against dairy yogurt (one-sample Wilcoxon signed-rank tests) are provided for plain yogurt alternatives in Supplementary Table S2, for plain yogurt-like alternatives in Supplementary Table S3, and for flavored yogurt alternatives in Supplementary Table S4.

Overall, substrate type was the dominant determinant of macronutrient profile. Soy-based products were the only category with a protein content comparable to dairy yogurt, while being significantly lower in energy, fat, saturated fat, carbohydrates and total sugars. Coconut-based products were significantly higher in energy, fat and saturated fat than dairy yogurt, yet among the lowest in protein. Among the remaining substrates, which were assessed descriptively owing to small sample sizes, oat-based products showed the highest carbohydrate content and almond-based products a comparatively low energy content, although both were low in protein. Salt was low and broadly comparable across all categories.

As fiber declaration is not mandatory and not available for all products, fiber was excluded from the statistical analysis and the corresponding descriptive data are provided for plain and flavored yogurt alternatives, as well as yogurt-like alternatives in Supplementary Table S5.

### 3.3 Micronutrient fortification and additives

#### 3.3.1 Fortification with micronutrients

Overall, a minority of all products (18.4 %; *n* = 18) were fortified with one or more micronutrients, 14 yogurt alternatives (17.1 %) and 4 yogurt-like alternatives (25 %). Fortification in the products was restricted to calcium (*n* = 18), vitamin D (*n* = 17), vitamin B_12_ (*n* = 17), and vitamin B_2_ (*n* = 12), with no other micronutrients reported. Fortification levels were identical across all products, and median micronutrient contents per 100 g were 120 mg calcium, 0.75 μg vitamin D, 0.38 μg vitamin B_12_, and 0.21 mg vitamin B_2_.

#### 3.3.2 Additives

The majority (89.8 %; *n* = 88) of all yogurt alternatives and yogurt-like alternatives contained at least one additive. The most frequently reported category comprised emulsifiers, gelling agents, stabilizers, or thickeners, present in 89.8 % of products, primarily in the form of starches, modified starches, pectins (E440), locust bean gum (E410), agar (E406), and guar gum (E412). Acidity regulators were detected in 13.3 % of products (*n* = 13), predominantly citric acid (E330), sodium citrates (E331), malic acid (E296), and calcium citrate (E333). Antioxidants were less common, with ascorbyl esters (E304) and tocopherol-rich extracts identified in a subset of 7 products.

In addition to the mentioned additives, a wide range of fruit and vegetable concentrates and extracts was used in more than half of all products (*n* = 52). Lemon juice concentrate and apple juice concentrate were the most common, alongside coloring concentrates such as beetroot concentrate and turmeric extract. Flavoring agents including “aroma” and “natural flavor” were also frequently present (*n* = 31). Furthermore, 83.3 % of all flavored products contained added sugars (total *n* = 55 products, *n* = 51 yogurt alternatives and *n* = 4 yogurt-like alternatives).

### 3.4 Micronutrient substitution modeling

The Swiss Food Pyramid recommends a daily intake of 175 g of yogurt (12). However, observed intake of yogurt in the Swiss population based on the national nutrition survey menuCH was 74.6 g per day (10). Table 2 summarizes the micronutrient intake based on this observed intake as well as under a scenario of complete replacement of dairy yogurt with coconut-, or soy-based alternatives. The proportion of the RDA achieved for each micronutrient is shown for all substitution scenarios. Supplementary Material S6 summarizes the same information for the recommended portion size of yogurt of 175 g as well as its replacement by soy- or coconut-based alternatives.

**Table 2:** Modeled daily micronutrient contribution from the observed intake portion of 74.6 g of yogurt (10), comparing plain dairy yogurt with substitutions using plain soy- and coconut-based yogurt alternatives. For each micronutrient, absolute intake per portion and the corresponding percentage of the recommended dietary allowance (RDA) for adults are presented, minimum and maximum ranges were reported to reflect sex-specific intake requirements (20).

Micronutrient	Unit	Dairy yogurt, plain, full-fat (Micronutrient intake in daily observed portion)	% of RDA (adults)	Soy yogurt alternative, plain (Micronutrient intake in daily observed portion)	% of RDA (adults)	Coconut yogurt alternative, plain (Micronutrient intake in daily observed portion)	% of RDA (adults)
Vitamin A activity, RE	μg	29.25	3.90–4.50	0.75	≤ 1.00	0.75	≤ 1.00
Vitamin B_1_ (Thiamine)	mg	0.02	1.79	0.01	≤ 1.00	0.00	≤ 1.00
Vitamin B_2_ (Riboflavin)	mg	0.14	8.44	0.00	≤ 1.00	0.00	≤ 1.00
Vitamin B_3_ (Niacin)	mg	0.07	≤ 1.00	0.04	≤ 1.00	0.08	0.58
Vitamin B_5_ (Pantothenic acid)	mg	0.25	4.95	0.03	≤ 1.00	0.02	≤ 1.00
Vitamin B_6_ (Pyridoxine)	mg	0.03	1.76–1.88	0.00	≤ 1.00	0.00	≤ 1.00
Vitamin B_9_ (Folate)	μg	3.75	1.14	13.95	4.23	0.38	≤ 1.00
Vitamin B_12_ (Cobalamin)	μg	0.23	5.63	0.00	≤ 1.00	0.00	≤ 1.00
Vitamin C (Ascorbic acid)	mg	1.50	1.36–1.58	0.19	≤ 1.00	0.00	≤ 1.00
Vitamin D (Calciferol)	μg	0.08	≤ 1.00	0.11	≤ 1.00	0.00	≤ 1.00
Vitamin E (α-Tocopherol)	mg	0.08	≤ 1.00	0.17	1.33–1.57	0.00	≤ 1.00
Potassium (K)	mg	127.50	3.64	82.50	2.36	69.00	1.97
Sodium (Na)	mg	36.75	1.84	25.50	1.28	18.75	≤ 1.00
Chloride (Cl)	mg	90.00	2.90	18.00	≤ 1.00	51.00	1.65
Calcium (Ca)	mg	105.00	10.50–11.05	10.50	1.05–1.11	3.75	≤ 1.00
Magnesium (Mg)	mg	9.00	2.57–3.00	12.75	3.64–4.25	7.28	2.08–2.43
Phosphorus (P)	mg	82.50	15.00	36.00	6.55	16.50	3.00
Iron (Fe)	mg	0.00	≤ 1.00	0.30	1.88–2.73	0.11	≤ 1.00
Iodine (I)	μg	12.00	8.00	n.d.	n.d.	n.d.	n.d.
Zinc (Zn)	mg	0.30	1.84–4.00	0.23	1.38–3.00	0.08	0.46–1.00

Median micronutrient values were obtained from the Swiss Food Composition Database (18).

## 4 Discussion

### 4.1 Broad presence of fermented plant-based yogurt alternatives in the Swiss market

We identified a wide range of fermented plant-based yogurt alternatives available in Switzerland, with soy-based products being the most prevalent, followed by coconut, mixed-substrate-, oat-, and almond-based options in plain and flavored varieties and substantial price variation. This heterogeneity suggests broad consumer availability of fermented plant-based yogurt alternatives. As plant-based dairy alternatives have lower environmental footprints than conventional dairy, and their substitution for animal-source dairy has been identified as a potential lever to reduce food system-related environmental impacts (7), the broad assortment observed in the Swiss retail market may facilitate consumer shifts toward more plant-forward diets.

### 4.2 Substrate-dependent macronutrient profiles of fermented plant-based yogurt alternatives

We showed that fermented plant-based yogurt alternatives differ substantially from dairy yogurt and should not be considered nutritionally equivalent. Substrate selection systematically influences energy, fat, total sugars, and protein composition. We found that coconut-based alternatives showed the highest energy and saturated fat contents across all substrate types, consistent with prior evidence (21). Importantly, their energy and saturated fat contents also exceeded those of dairy yogurt. In contrast, soy- and almond-based products contained less energy than dairy yogurt and displayed more favorable fat profiles. Within each substrate, flavored varieties consistently contained more energy and total sugars than their plain counterparts, largely from added sugars.

In the Swiss population, where over 40 % of adults have overweight or obesity and prevalence continues to rise alongside increased cardiometabolic and mental health risks (22), replacing dairy yogurt with higher-energy plant-based alternatives, such as coconut-based or flavored products, may counteract dietary recommendations by increasing energy, saturated fat, and total sugars intake when consumed in comparable portions. Conversely, plain soy- and almond-based alternatives may better align with dietary strategies aimed at reducing saturated fat intake and supporting cardiometabolic health.

Protein adequacy warrants particular attention, as one fifth of Swiss adults do not meet current protein recommendations (23). We found that only soy-based alternatives approached the protein content of dairy yogurt. This aligns with previous evidence identifying soy-based products as the highest-protein fermented plant-based yogurt alternatives (24). All other substrate types fell below dairy yogurt in protein content. Moreover, the literature also indicates that plant-based options generally provide not only lower total protein content but also less favorable amino acid profiles and reduced protein digestibility (24). Thus, beyond nutrient quantities, differences in the food matrix may further limit equivalence. Given the reduced protein digestibility and mineral bioavailability reported for these products (24), their distinct matrices mean that similar label values need not correspond to similar nutrient delivery. Compositional data alone therefore cannot establish nutritional equivalence with dairy.

### 4.3 Mineral intake vulnerabilities associated with dairy-to-plant yogurt substitution

In our modeling, dairy yogurt proved to be a relevant source of calcium and contributed approximately 11–26 % of the RDA, depending on the intake levels. In contrast, soy- and coconut-based yogurt supplied 3 % or less of calcium. Substituting dairy yogurt with unfortified plant-based alternatives may therefore exacerbate existing calcium inadequacy, which is already a concern in Switzerland and particularly pronounced among individuals adhering to vegan diets (25, 26). This is clinically relevant given the well-established associations between insufficient calcium intake, reduced bone mineral density, and increased fracture risk (27).

We further observed that fermented plant-based yogurt alternatives contain substantially less phosphorus than dairy yogurt. Given that phosphorus intake is generally adequate in the Swiss population (26), this lower contribution is unlikely to increase deficiency risk. However, mineral balance warrants consideration. Dietary patterns characterized by relatively high phosphorus intake in the context of low calcium can disrupt calcium-phosphorus homeostasis and negatively affect bone health (28). Accordingly, replacing calcium-rich dairy products with lower-calcium plant-based alternatives may unfavorably alter the dietary calcium-to-phosphorus ratio.

Despite the widespread use of iodized salt, iodine intake in Switzerland remains below recommendations, with dairy products and iodized salt as the primary dietary sources (26, 29). Our modeling indicated that dairy yogurt provided approximately 8–19 % of the RDA for iodine, depending on the intake levels, while soy- and coconut-based alternatives contained no detectable iodine. Given that pregnant and lactating women, infants, children, and women of reproductive age are specifically identified as target populations at risk of iodine deficiency (29), replacing dairy yogurt with plant-based alternatives may result in a meaningful reduction in dietary iodine and increase the risk of inadequacy.

### 4.4 Vitamin intake shifts under substitution with unfortified fermented plant-based yogurt alternatives

The contribution of yogurt to B-vitamin intake in our modeling varied substantially by product type and fortification status. For vitamin B_2_, dairy yogurt represented a meaningful source, contributing up to 20 % at recommended intake levels. In contrast, soy- and coconut-based fermented yogurt alternatives provided negligible amounts. Consistent with expectations for plant-based products, unfortified soy- and coconut-based fermented yogurt alternatives contributed no measurable vitamin B_12_. Dairy yogurt in contrast supplied 6–13 % of the RDA depending on the yogurt intake level. While fortified products reached levels comparable to dairy yogurt for vitamin B_2_ and even exceeded levels for vitamin B_12_, the limited frequency of fortification suggests that the majority of consumers will not ingest meaningful amounts of those vitamins from plant-based yogurt alternatives. For vitamin B_5_, dairy yogurt contributed moderate amounts (5–12 % of the RDA), whereas soy- and coconut-based alternatives supplied 1 % or less. Although vitamin B_5_ deficiency is uncommon in Switzerland, intake among women falls below recommendations (26), which should be considered when substituting for dairy.

Unlike other B-vitamins, folate intake was higher from fermented soy yogurt alternatives than from dairy yogurt, providing almost 10 % at recommended intake levels compared to less than 3 % from dairy yogurt. Thus, soy-based alternatives may modestly improve folate intake compared to dairy yogurt and fermented coconut yogurt alternatives.

Vitamin A status in Switzerland is generally adequate (26), and deficiency is uncommon in plant-based diets (30). In our modeling, dairy yogurt contributed 4–11 % vitamin A depending on the intake levels, whereas soy and coconut yogurt alternatives contributed marginally. Although fermented plant-based yogurt alternatives contain markedly lower vitamin A levels, replacing dairy yogurt with these products is unlikely to meaningfully affect vitamin A adequacy at the population level in Switzerland.

For the remaining micronutrients (vitamins B1, B3, B6, C, D, E and zinc, potassium, sodium, chloride, magnesium, and iron), all yogurt types contributed only small proportions of daily requirements (< 10 % RDA in all scenarios). Dairy yogurt generally supplied higher amounts of vitamins B1, B3, B6, C and zinc, potassium, sodium, and chloride. Conversely, soy-based alternatives provided marginally higher levels of vitamin D, vitamin E, magnesium and iron than dairy yogurt.

### 4.5 Limited micronutrient fortification of fermented plant-based yogurt alternatives in Switzerland

We found substantial differences in micronutrient provision between dairy yogurt and unfortified plant-based alternatives. Depending on intake level, dairy yogurt contributes ≥10 % of the RDA for calcium, iodine, phosphorus, vitamin A, and selected B-vitamins (B_2_, B5, and B1_2_), whereas unfortified plant-based alternatives contribute minimally across these nutrients. Beyond concentration differences, data from European products demonstrated that key minerals in plant-based products are also less bioavailable due to phytate- and saponin-rich plant matrices, particularly in soy-based formulations (24), meaning that label-derived values may overestimate the effective nutritional contribution.

Despite these challenges, we identified that only 18.4 % of all products were fortified, which contrasts with fortification patterns reported in other countries. In the United Kingdom, a market survey found that 63.6 % of fermented plant-based yogurt alternatives were fortified with one or more micronutrients (31). Similarly, Swedish market data showed fortification rates of over 80 % for vitamin D, over 70 % with calcium and vitamin B1_2_, nearly 60 % for vitamin B_2_, and almost 20 % for iodine (21). Where fortification is present on the Swiss market, it is largely limited to vitamin D, calcium, vitamin B_2_, and vitamin B1_2_, nutrients for which fortified alternatives can approach dairy yogurt levels, and in the case of vitamin B1_2_, even exceed them. However, fortification practices in Switzerland are not extended to micronutrients such as iodine or vitamin B5, meaning residual gaps persist for those micronutrients even when consumers choose fortified products.

Against this background, the limited fortification of fermented plant-based yogurt alternatives on the Swiss market indicates that replacing dairy yogurt with predominantly unfortified alternatives may elevate the risk of micronutrient inadequacy. Even where fortified products are chosen, gaps remain for nutrients outside the scope of current fortification practices and may require compensation through other dietary sources.

### 4.6 Ultra-processing and consumer perception of plant-based yogurt alternatives

From a food processing perspective, the formulation characteristics of the surveyed products warrant consideration. According to the NOVA classification system, ultra-processed foods (UPFs) can be defined as foods made from ingredients not typically used in a home kitchen (32). Given that nearly 90 % of the surveyed products contained additives, including emulsifiers, thickeners, and acidity regulators, which fall outside conventional domestic food preparation, a substantial proportion of the plant-based alternatives assessed here may qualify as UPFs. This carries public health relevance, as growing evidence links higher UPF intake to adverse cardiometabolic outcomes, mental health risks, and higher all-cause mortality (33).

Beyond the degree of processing, we identified several additional product characteristics that may challenge consumer acceptance. Given that Swiss consumers source approximately 84 % of their dairy yogurt purchases from domestic producers (34), the prevalence of products originating from outside of Switzerland produced with imported substrates observed in more than half of the plant-based yogurt alternatives surveyed may represent a barrier to how these products are perceived in terms of local authenticity and regional identity. Together with the high prevalence of added sugars in flavored products and the frequent use of industrial additives, these characteristics may affect how consumers perceive their naturalness. Perceived naturalness is a major determinant of consumer acceptance and is primarily evaluated based on product origin and locality, production methods and characteristics of the final product, with products perceived as less natural being less likely to be accepted by consumers (35). The formulation profile observed across the Swiss market for plant-based fermented yogurt alternatives may therefore represent barriers to broader consumer uptake, despite the well-documented environmental advantages of plant-based alternatives over dairy.

### 4.7 Strengths, limitations and future perspectives

This market survey covered major Swiss retail channels, including supermarkets, discounters, online platforms, and selected specialty stores, providing a comprehensive overview of fermented plant-based yogurt alternatives available to consumers. Using an operational definition based on the presence of viable microorganisms, we focused on fermented yogurt alternatives and yogurt-like products with live cultures. To our knowledge, this is among the first studies to combine detailed Swiss market data with nationally representative consumption data (menuCH) and Swiss dietary reference values to assess macro- and micronutrient provision of fermented plant-based yogurt alternatives.

Several limitations should be acknowledged. Nutrient and fortification data were derived from product labels and food composition databases without laboratory verification. As the substitution modeling relied on median micronutrient values from food composition databases, those values cannot capture potential between-product variability arising from agricultural practices, raw-material composition or processing. Combined with the lack of laboratory verification, this means our estimates represent typical rather than product-specific micronutrient provision, and the contribution of any individual product may deviate from the modeled value. Moreover, the analysis did not capture fermentation-related functional properties. Future research should integrate market audits with microbiological and functional profiling to better reflect the health-relevant properties of fermented plant-based foods.

Our analysis reflects a cross-sectional snapshot of the Swiss retail market in 2025. Product availability and fortification practices may change over time or differ across countries. Substitution modeling assumed a 1:1 replacement by weight of plain, unfortified products and did not account for dietary context or nutrient bioavailability, potentially underestimating intakes among consumers choosing fortified products, while potentially overestimating equivalence in bioavailable nutrient intake between dairy and fermented plant-based yogurt alternatives. Further, our modeling compared intakes against RDAs for the general adult population and did not account for the higher or distinct requirements of specific groups such as pregnant and breastfeeding women, children, and older adults. This is particularly relevant because plant-based diets appear to be predominantly adopted by young women (36), whose nutritional requirements are elevated during pregnancy and lactation (20). Thus, our findings consequently cannot be assumed to hold for pregnant or breastfeeding women, children, or older adults, who warrant separate evaluation.

Overall, the Swiss market offers a broad and expanding range of fermented plant-based yogurt alternatives that could facilitate shifts toward more plant-based dietary patterns. At the same time, we observed formulation characteristics, including the widespread use of additives, imported substrates and added sugars, that may raise concerns regarding consumer acceptance and alignment with dietary guidance that emphasizes minimally processed plant foods.

Because fortification is country-specific, the micronutrient gaps modeled here would narrow in markets where fortification is more common, such as the United Kingdom and Sweden (21, 31), and the importance of each gap depends on local baseline adequacy. Although evidence on fortification practices in plant-based yogurt alternatives from neighboring countries is limited, studies on plant-based drinks in Germany report similarly low levels of fortification (37), suggesting comparable trends across Central European food environments. The substrate-driven macronutrient differences, by contrast, are grounded in raw-material composition and are therefore more likely to transfer across settings. Among the predominant formulations, soy-based products most closely resembled dairy yogurt in protein content with lower saturated fat, whereas coconut-based products were more energy-dense and higher in saturated fat. Flavored products consistently increased total sugar content across all substrates.

Substitution modeling further demonstrated that unfortified replacement of dairy yogurt with plant-based alternatives leads to pronounced reductions in calcium, iodine, vitamin B_2_ and vitamin B_12_ intake, with additional decreases in phosphorus, zinc, vitamin A and vitamin B5, indicating that these micronutrients require particular attention when dairy substitution is promoted. An exception was folate, for which soy-based yogurt alternatives provided higher contributions than dairy yogurt. The scope for offsetting micronutrient gaps through fortification is itself constrained, as Regulation (EU) 2018/848 and the equivalent Swiss ordinance permit micronutrient addition to organic foods only where legally required, certified-organic products are barred from voluntary fortification (38, 39), a limit that applies across the EU and Switzerland alike. In Switzerland, where fortification of fermented plant-based yogurt alternatives is in any case comparatively limited, these findings indicate that such products are not nutritionally interchangeable with dairy yogurt. Achieving nutritionally adequate plant-forward dietary transitions will therefore require informed product selection, more systematic fortification practices, and, where appropriate, partial rather than complete substitution strategies.

## Data Availability

The original contributions presented in the study are included in the article/Supplementary material, further inquiries can be directed to the corresponding author.
