# Supplementary material for: Fermented plant-based yogurt alternatives in Switzerland: nutritional adequacy and fortification practices
Source: Front Nutr. 2026 Aug 11;13:1899857. doi: 10.3389/fnut.2026.1899857 (PMC13503149; doi:10.3389/fnut.2026.1899857)
Supplement: Supplementary file 1 [file Supplementary_Material.docx]

**Supplementary material**

**Fermented plant-based yogurt and yogurt-like alternatives identified in the Swiss market**

**Supplementary table S1.** List of fermented plant-based yogurt and yogurt-like alternatives identified in the Swiss market, with nutrient information extracted from the product nutrition labels.

| Name | Description (Production location) | Brand | Ingredients | Energy (kcal/  100g) | Fat  (g/100g) | Saturated Fat (g/100g) | Carbohydrates (g/100g) | Total sugars (g/100g) | Fiber  (g/100g) | Protein  (g/100g) | Vitamin supplementation |
| --- | --- | --- | --- | --- | --- | --- | --- | --- | --- | --- | --- |
| Alnatura Kokos Natur vegan | Yogurt alternative based on coconut, nature (Germany) | Alnatura | Coconut milk 95% (water, coconut pulp 24%), tapioca starch, stabilizer: locust bean gum, sea salt, lactic acid bacteria cultures | 95 | 7.7 | 7.1 | 5.4 | 0.8 | 0.5 | 0.7 |  |
| Alnatura Mandel Natur vegan | Yogurt alternative based on almond, nature (Germany) | Alnatura | Almond drink 95% (water, almonds 6%), tapioca starch, stabilizer: locust bean gum, sea salt, lactic acid bacteria cultures | 52 | 3.3 | 0.3 | 5 | 0.25 | 1.3 | 0.25 |  |
| Alnatura Soja Natur vegan | Yogurt alternative based on soy, nature (France) | Alnatura | Soy drink 99% (water, dehulled soybeans 9.2%), lactic acid bacteria cultures | 45 | 2.9 | 0.4 | 0.25 | 0.25 | 0.25 | 4.8 |  |
| alpro Heidelbeere | Yogurt alternative based on soy, with blueberries (Belgium) | alpro | Soy base (water, dehulled soybeans 9.8%), sugar, blueberry 5.7%, tricalcium citrate, stabilizer (pectins), acidity regulators (citric acid, sodium citrates), natural flavoring, blueberry extract, carrot extract, sea salt, antioxidants (tocopherol-rich extracts, fatty acid esters of ascorbic acid), vitamins (B2, B12, D2), yogurt cultures (Streptococcus thermophilus, Lactobacillus bulgaricus) | 70 | 2.2 | 0.4 | 8.2 | 7.9 | 1 | 3.7 | Calcium (120mg/100g), Vit D (0.75µg/100g), Vit B12 (0.38µg/100g), Vit B2 (0.21mg/100g) |
| alpro Himbeere Apfel ohne Zuckerzusatz | Yogurt alternative based on soy, with apples and raspberries (Belgium) | alpro | Soy base (water, dehulled soybeans 9.5%), raspberry mixture 10.8% (juice from concentrate 5.8%, fruit 5%), apple juice from concentrate 7.6%, tricalcium citrate, stabilizer (pectins), natural flavoring, maize starch, acidity regulators (citric acid, sodium citrates), sea salt, carrot extract, antioxidants (tocopherol-rich extracts, fatty acid esters of ascorbic acid), vitamins (B2, B12, D2), yogurt cultures (Streptococcus thermophilus, Lactobacillus bulgaricus) | 58 | 1.9 | 0.3 | 4.8 | 4.4 | 1.3 | 3.6 | Calcium (120mg/100g), Vit D (0.75µg/100g), Vit B12 (0.38µg/100g), Vit B2 (0.21mg/100g) |
| alpro Lemon Cheesecake | Yogurt alternative based on soy, with cheesecake aroma (Belgium) | alpro | Soy base 86.5% (water, dehulled soybeans 9.9%), sugar, lemon juice from concentrate 1.7%, lemon 1.1%, glucose–fructose syrup, calcium (tricalcium citrate), stabilizer (pectins), acidity regulators (citric acid, sodium citrates), modified starch, sea salt, flavoring, antioxidants (tocopherol-rich extracts, fatty acid esters of ascorbic acid), vitamins (B2, D2, B12), yogurt cultures (Streptococcus thermophilus, Lactobacillus bulgaricus) | 71 | 2.2 | 0.4 | 8.7 | 8.4 | 0.8 | 3.7 | Calcium (120mg/100g), Vit D (0.75µg/100g), Vit B12 (0.38µg/100g), Vit B2 (0.21mg/100g) |
| alpro Mango ohne Zuckerzusatz | Yogurt alternative based on soy, with mango (Belgium) | alpro | Soy base (water, dehulled soybeans 9.7%), mango mixture 17.7% (juice from concentrate 11%, fruit 6.7%), tricalcium citrate, stabilizer (pectins), acidity regulators (sodium citrates, citric acid), natural flavoring, modified starch, sea salt, antioxidants (tocopherol-rich extracts, fatty acid esters of ascorbic acid), pumpkin extract, carrot extract, vitamins (B2, B12, D2), yogurt cultures (Streptococcus thermophilus, Lactobacillus bulgaricus) | 61 | 2 | 0.4 | 5.8 | 5.6 | 1 | 3.7 | Calcium (120mg/100g), Vit D (0.75µg/100g), Vit B12 (0.38µg/100g), Vit B2 (0.21mg/100g) |
| alpro Natur ohne Zucker | Yogurt alternative based on soy, nature (Belgium) | alpro | Soy base (water, dehulled soybeans 10.7%), tricalcium citrate, acidity regulators (sodium citrates, citric acid), stabilizer (pectins), natural flavoring, sea salt, antioxidant (tocopherol-rich extracts, fatty acid esters of ascorbic acid), vitamins (B12, D2), yogurt cultures (Streptococcus thermophilus, Lactobacillus bulgaricus). | 42 | 2.3 | 0.4 | 0 | 0 | 0.9 | 4 | Calcium (120mg/100g), Vit D (0.75µg/100g), Vit B12 (0.38µg/100g) |
| alpro PLANT PROTEIN Mango-Banane | Quark-like alternative based on soy, with mango and banana (Belgium) | alpro | Soy base 85.1% (water, dehulled soybeans 9.8%), sugar, soy protein isolate, mango–banana mix 2.0% (mango 1.5%, banana 0.5%), faba bean protein, calcium (tricalcium citrate), stabilizer (pectins), natural flavorings, carrot and pumpkin concentrate, acidity regulators (citric acid, sodium citrates), vitamins (B12, D2), yogurt cultures (Streptococcus thermophilus, Lactobacillus bulgaricus) | 82 | 2.2 | 0.4 | 7.2 | 7 | 1 | 7.5 | Calcium (120mg/100g), Vit D (0.75µg/100g), Vit B12 (0.38µg/100g) |
| alpro PLANT PROTEIN Rote Früchte | Quark-like alternative based on soy, with berries (Belgium) | alpro | Soy base 85% (water, dehulled soybeans 9.8%), sugar, soy protein isolate, strawberry–red currant–raspberry mix 2% (strawberry 0.8%, red currant 0.8%, raspberry 0.4%), faba bean protein, calcium (tricalcium citrate), stabilizer (pectins), natural flavorings, modified starch, coloring black carrot concentrate, acidity regulators (citric acid, sodium citrates), vitamins (B12, D2), yogurt cultures (Streptococcus thermophilus, Lactobacillus bulgaricus) | 81 | 2.2 | 0.4 | 7 | 6.8 | 1.1 | 7.5 | Calcium (120mg/100g), Vit D (0.75µg/100g), Vit B12 (0.38µg/100g) |
| alpro Skyr Joghurtalternative ohne Zucker | Skyr-like alternative based on soy, nature (Belgium) | alpro | Soy base (water, dehulled soybeans 15.7%), stabilizer (pectins), tricalcium citrate, acidity regulators (sodium citrates, citric acid), natural flavoring, antioxidants (tocopherol-rich extracts, fatty acid esters of ascorbic acid), sea salt, vitamins (B12, D2), yogurt cultures (Streptococcus thermophilus, Lactobacillus bulgaricus) | 61 | 3.3 | 0.6 | 0 | 0 | 1.3 | 5.8 | Calcium (120mg/100g), Vit D (0.75µg/100g), Vit B12 (0.38µg/100g) |
| alpro Skyr Joghurtalternative Vanille | Skyr-like alternative based on soy, with vanilla (Belgium) | alpro | Soy base (water, dehulled soybeans 13.9%), sugar, stabilizer (pectins), tricalcium phosphate, acidity regulators (sodium citrates, malic acid, citric acid), natural flavoring, antioxidants (tocopherol-rich extracts, fatty acid esters of ascorbic acid), sea salt, vanilla powder 0.02%, natural vanilla flavoring with other natural flavorings, carrot extract, vitamins (B2, B12, D2), yogurt cultures (Streptococcus thermophilus, Lactobacillus bulgaricus) | 77 | 3 | 0.5 | 6.8 | 6.5 | 1.1 | 5.1 | Calcium (120mg/100g), Vit D (0.75µg/100g), Vit B12 (0.38µg/100g), Vit B2 (0.21mg/100g) |
| ANDROS Natur mit Kokos | Yogurt alternative based on coconut, nature (France) | Andros | Reduced-fat coconut milk 92% (water, coconut extract), modified tapioca starch, pea protein, calcium phosphates, salt, cultures | 77 | 4.8 | 4.6 | 7.7 | 0.5 |  | 0.5 | Calcium (120mg/100g) |
| beleaf HASELNUSS | Yogurt alternative based on mixed substrate, with hazelnut (Switzerland) | beleaf | Water, sugar, coconut 4.5%, soy 4%, hazelnuts 2%, hazelnut paste 2%, faba bean protein, pea protein, almond paste, starch, calcium phosphate, lemon juice concentrate, salt, flavorings, vitamins (D, B2, B12), yogurt cultures | 107 | 5.6 | 1.8 | 9.9 | 8.3 |  | 4 | Calcium (120mg/100g), Vit D (0.75µg/100g), Vit B12 (0.38µg/100g), Vit B2 (0.21mg/100g) |
| beleaf NATURE ungesüsst | Yogurt alternative based on mixed substrate, nature (Switzerland) | beleaf | Water, coconut 5%, soy 5%, faba bean protein, pea protein, starch, calcium phosphate, salt, flavorings, vitamins (D, B2, B12), yogurt cultures | 57 | 3.5 | 2 | 2.1 | 0.8 |  | 4 | Calcium (120mg/100g), Vit D (0.75µg/100g), Vit B12 (0.38µg/100g), Vit B2 (0.21mg/100g) |
| beleaf STRACCIATELLA | Yogurt alternative based on mixed substrate, with stracciatella (Switzerland) | beleaf | Water, sugar, coconut 4.5%, soy 4%, chocolate chips 2.5%, faba bean protein, pea protein, modified starch, thickener (pectin), calcium phosphate, lemon juice concentrate, salt, natural flavorings, vitamins (D, B2, B12), yogurt cultures | 105 | 4.2 | 2.5 | 13 | 12 |  | 3.3 | Calcium (120mg/100g), Vit D (0.75µg/100g), Vit B12 (0.38µg/100g), Vit B2 (0.21mg/100g) |
| beleaf WALDBEERE | Yogurt alternative based on mixed substrate, with berries (Switzerland) | beleaf | Water, sugar, berries 6% (elderberries 2%, blackberries 1%, strawberries 1%, blueberries 1%, raspberries 1%), coconut 4.5%, soy 4%, starch, faba bean protein, pea protein, calcium phosphate, lemon juice concentrate, beetroot juice concentrate, salt, flavorings, vitamins (D, B2, B12), yogurt cultures | 82 | 2.9 | 1.7 | 10 | 8.2 |  | 3.4 | Calcium (120mg/100g), Vit D (0.75µg/100g), Vit B12 (0.38µg/100g), Vit B2 (0.21mg/100g) |
| BIEDERMANN BIO VEGAN HAFER Nature | Yogurt alternative based on oat, nature (Switzerland) | Biedermann | Oat drink (water, oats 12%), coconut oil, maize starch, guar gum, vegan yogurt cultures | 95 | 5.4 | 4.6 | 11.4 | 2.8 |  | 0.2 |  |
| BIEDERMANN BIO VEGAN HEIDELBEERE COCO ORGANIC | Yogurt alternative based on coconut, with blueberries (Switzerland) | Biedermann | Light coconut milk 78% (water, coconut extract 21%), blueberries 8%, raw cane sugar, maize starch, rice starch, gelling agent: agar-agar, lemon juice concentrate, lactic acid bacteria | 120 | 7.2 | 6.8 | 13 | 9.3 |  | 0.8 |  |
| BIEDERMANN BIO VEGAN MANGO COCO ORGANIC | Yogurt alternative based on coconut, with mango (Switzerland) | Biedermann | Light coconut milk 78% (water, coconut extract 21%), raw cane sugar, mango 7%, maize starch, rice starch, gelling agent: agar-agar, lemon juice concentrate, lactic acid bacteria | 125 | 7.2 | 6.8 | 14 | 11 |  | 0.8 |  |
| BIEDERMANN BIO VEGAN Nature COCO ORGANIC | Yogurt alternative based on coconut, nature (Switzerland) | Biedermann | Light coconut milk 96% (water, coconut extract 26%), maize starch, gelling agent: agar-agar, lemon juice concentrate, lactic acid bacteria cultures | 101 | 8.9 | 8.4 | 4.4 | 1 |  | 0.9 |  |
| BioMandel-Alternative zu Joghurt | Yogurt alternative based on almond, nature (Switzerland) | Soyana | Water, 12% almonds, thickener: agar-agar, pectin, vegan lactic acid bacteria cultures | 53 | 6.7 | 0.5 | 2.8 | 0.8 | 1.9 | 2.5 |  |
| COCONUT HARVEST MOON CHOCOLATE | Yogurt alternative based on coconut, with chocolate (Austria) | Harvest Moon | Coconut milk 77% (coconut, water), coconut blossom nectar, cocoa 7%, agave, lemon, tapioca starch, vegan yogurt cultures (S. thermophilus, L. delbrueckii subsp. bulgaricus) | 121 | 6.1 | 5.5 | 13.2 | 8.9 |  | 1.7 |  |
| COCONUT HARVEST MOON DARK BERRY | Yogurt alternative based on coconut, with berries (Austria) | Harvest Moon | Coconut milk 64% (coconut, water), blueberry, grape, tapioca starch, blackberry, banana, blackcurrant, apple, agave, vegan yogurt cultures (S. thermophilus, L. delbrueckii subsp. bulgaricus) | 105 | 5.7 | 5.3 | 11.8 | 7.3 |  | 0.7 |  |
| COCONUT HARVEST MOON MANGO MARACUJA | Yogurt alternative based on coconut, with mango and maracuja (Austria) | Harvest Moon | Coconut milk 80% (coconut, water), mango 10%, raw cane sugar, starch, Bourbon vanilla, lemon, vegan yogurt cultures (S. thermophilus, L. delbrueckii subsp. bulgaricus) | 95 | 5.9 | 5.5 | 9.4 | 4.9 |  | 0.6 |  |
| COCONUT HARVEST MOON NATUR | Yogurt alternative based on coconut, nature (Austria) | Harvest Moon | Coconut milk 99% (coconut, water), tapioca starch, vegan yogurt cultures (S. thermophilus, L. delbrueckii subsp. bulgaricus) | 97 | 8 | 7.7 | 5.3 | 0.6 |  | 0.7 |  |
| COCONUT HARVEST MOON STRACCIATELLA | Yogurt alternative based on coconut, with stracciatella (Austria) | Harvest Moon | Coconut milk 83% (coconut, water), raw cane sugar, maize starch, chocolate pieces 2% (cocoa butter, cocoa mass, raw cane sugar), tapioca starch, thickener: locust bean gum, Bourbon vanilla, lemon, vegan yogurt cultures | 138 | 7.9 | 7.1 | 15.5 | 10.4 |  | 0.6 |  |
| COCONUT HARVEST MOON STRAWBERRY | Yogurt alternative based on coconut, with strawberries (Austria) | Harvest Moon | Coconut milk 63% (coconut, water), strawberry 13%, grape, raspberry, tapioca starch, banana, lime, beetroot, vegan yogurt cultures (S. thermophilus, L. delbrueckii subsp. bulgaricus) | 122 | 7.6 | 6.6 | 12.5 | 8.7 |  | 0.8 |  |
| COCONUT HARVEST MOON VANILLE | Yogurt alternative based on coconut, with vanille (Austria) | Harvest Moon | Coconut milk 90% (coconut, water), agave, maize starch, tapioca starch, Bourbon vanilla, lemon, thickener: locust bean gum, vegan yogurt cultures (S. thermophilus, L. delbrueckii subsp. bulgaricus) | 109 | 7.9 | 7.7 | 9.3 | 4.4 |  | 0.6 |  |
| HAFER-OAT HARVEST MOON BLUEBERRY VANILLA | Yogurt alternative based on oat, with blueberries and vanilla (Austria) | Harvest Moon | Oat drink 74% (water, oats), blueberries 7%, grape, rice, maize starch, coconut oil, tapioca starch, Bourbon vanilla, elderberry, lemon, sea salt, thickener: locust bean gum, vegan yogurt cultures (S. thermophilus, L. delbrueckii subsp. bulgaricus). | 92 | 3 | 2.6 | 15.3 | 8.9 |  | 0.2 |  |
| HAFER-OAT HARVEST MOON MANGO MARACUJA | Yogurt alternative based on oat, with mango and maracuja (Austria) | Harvest Moon | Oat drink 74% (water, oats), peach, grape, maize starch, rice, coconut oil, passion fruit 3%, mango, tapioca starch, lemon, carrot, sea salt, thickener: locust bean gum, vegan yogurt cultures (S. thermophilus, L. delbrueckii subsp. bulgaricus). | 88 | 2.9 | 2.5 | 14.9 | 8.5 |  | 0.3 |  |
| Karma Bio Fairtrade Kokos Mango | Yogurt alternative based on coconut, with mango (Switzerland) | Karma | Water, coconut milk 26% (coconut, water), mango purée 9%, sugar 6.3%, starch (waxy maize, cassava), lemon juice concentrate, thickener (E410), salt | 104 | 6.4 | 6 | 11 | 7.6 | 0 | 0.7 |  |
| Karma Bio Fairtrade Kokos Nature | Yogurt alternative based on coconut, nature (Switzerland) | Karma | Water, coconut milk 33% (coconut [Sri Lanka], water), waxy maize starch, thickener (E410), salt | 92 | 7.9 | 7.5 | 4.4 | 0 | 0 | 0.8 |  |
| Karma Bio Fairtrade Kokos Vanile | Yogurt alternative based on coconut, with vanille (Switzerland) | Karma | Water, coconut milk 27% (coconut, water), sugar 7.2%, waxy maize starch, lemon juice concentrate, thickener (E410), vanilla extract 0.07%, vanilla seeds 0.06%, salt | 105 | 6.5 | 6.2 | 11 | 7.4 | 0 | 0.7 |  |
| Karma Bio Fairtrade Soja Schokolade | Yogurt alternative based on soy, with chocolate (Switzerland) | Karma | Water, sugar 10.6% (Switzerland), dehulled soybeans 6%, cassava starch, dark chocolate 1.8% (cocoa mass 1.2%, cane sugar, cocoa butter 0.1%), low-fat cocoa powder 1.2%, lemon juice concentrate | 93 | 2.8 | 0.9 | 13 | 11 | 1 | 3.5 |  |
| Karma Bio Soja Waldbeeren | Yogurt alternative based on soy, with berries (Switzerland) | Karma | Water, sugar 6.3% (Switzerland), dehulled soybeans 6%, strawberries 6%, raspberries 3.5%, blackberries 3.5%, currants 3%, cassava starch | 71 | 1.9 | 0.3 | 9.8 | 7.6 | 1.1 | 3.1 |  |
| Karma Bio Sojo Natur ohne Zuckerzusatz | Yogurt alternative based on soy, nature (Switzerland) | Karma | Water, dehulled soybeans 8%, cassava starch | 47 | 2.5 | 0.4 | 1.8 | 0 | 0.6 | 3.9 |  |
| My Vay Bio Hafercreme | Yogurt alternative based on oat, nature (Austria) | My Vay | Water, 10% oats, coconut oil, starch, thickener: guar gum, salt, lactic acid cultures | 77 | 3.5 | 2.7 | 11 | 3.2 |  | 0.25 |  |
| My Vay Bio Kokoscreme | Yogurt alternative based on coconut, nature (Germany) | My Vay | Water, 29% coconut cream (coconut extract, water), starch, thickener: guar gum, lactic acid cultures | 95 | 7.7 | 7.1 | 5 | 0.6 |  | 1 |  |
| My Vay Bio Kokoscreme Heidelbeere | Yogurt alternative based on coconut, with blueberries (Swirzerland) | My Vay | Light coconut milk 80% (water, coconut extract 21%), raw cane sugar, blueberries 8%, maize starch, rice starch, gelling agent: agar-agar, lemon juice concentrate, lactic acid bacteria | 117 | 7.2 | 6.8 | 12 | 8.7 |  | 0.8 |  |
| My Vay Bio Kokoscreme Vanille | Yogurt alternative based on coconut, with vanille (Switzerland) | My Vay | Light coconut milk 88% (water, coconut extract 21%), raw cane sugar, maize starch, vanilla extract 0.2%, vanilla seeds, gelling agent: agar-agar, lemon juice concentrate, lactic acid bacteria | 114 | 7 | 6.6 | 11 | 7.7 |  | 0.7 |  |
| My Vay Bio Mandelcreme | Yogurt alternative based on almond, nature (Austria) | My Vay | Water, 6% almonds, starch, thickener: guar gum, salt, lactic acid cultures | 55 | 3.4 | 0.4 | 4.4 | 0.25 |  | 1.6 |  |
| My Vay Sojajogurt Heidelbeere | Yogurt alternative based on soy, with blueberries (Germany) | My Vay | Water, soybeans 11.6%, sugar, blueberries 5.5%, modified starch, blueberry juice from concentrate 1.1%, apple extract, starch, acidity regulators (citric acid, sodium citrates, malic acid), coloring plant concentrate (carrot), calcium salts of orthophosphoric acid, natural flavoring, calcium salts of citric acid, sea salt, thickener (locust bean gum), cyanocobalamin (vitamin B12), ergocalciferol (vitamin D2), riboflavin (vitamin B2), yogurt cultures | 74 | 2 | 0.3 | 10 | 8.2 |  | 3.6 | Calcium (120mg/100g), Vit D (0.75µg/100g), Vit B12 (0.38µg/100g), Vit B2 (0.21mg/100g) |
| My Vay Sojajogurt Mango | Yogurt alternative based on soy, with mango (Germany) | My Vay | Water, soybeans 11.6%, sugar, mango 5.1%, mango purée from concentrate 1.6%, modified starch, starch, calcium salts of orthophosphoric acid, acidity regulators (citric acid, sodium citrates), natural flavoring, calcium salts of citric acid, coloring plant concentrate (carrot), sea salt, thickener (locust bean gum), cyanocobalamin (vitamin B12), ergocalciferol (vitamin D2), riboflavin (vitamin B2), yogurt cultures | 71 | 2 | 0.3 | 9.4 | 7.9 |  | 3.6 | Calcium (120mg/100g), Vit D (0.75µg/100g), Vit B12 (0.38µg/100g), Vit B2 (0.21mg/100g) |
| My Vay Sojajogurt Natur ungesüsst | Yogurt alternative based on soy, nature (Germany) | My Vay | Water, 14% soybeans, modified starch, starch, calcium salts of orthophosphoric acid, sea salt, natural flavoring, cyanocobalamin (vitamin B12), ergocalciferol (vitamin D2), yogurt cultures | 46 | 2.3 | 0.4 | 1.8 | 0 |  | 4.2 | Calcium (120mg/100g), Vit D (0.75µg/100g), Vit B12 (0.38µg/100g) |
| My Vay Sojajogurt Vanille | Yogurt alternative based on soy, with vanilla (Germany) | My Vay | Water, soybeans 11.6%, sugar, modified starch, starch, calcium salts of orthophosphoric acid, natural flavoring, acidity regulators (citric acid, sodium citrates), calcium salts of citric acid, coloring plant concentrate (carrot), sea salt, thickener (locust bean gum), ground vanilla pods, cyanocobalamin (vitamin B12), ergocalciferol (vitamin D2), riboflavin (vitamin B2), yogurt cultures | 70 | 2 | 0.3 | 9.3 | 7.6 |  | 3.5 | Calcium (120mg/100g), Vit D (0.75µg/100g), Vit B12 (0.38µg/100g), Vit B2 (0.21mg/100g) |
| Nattosana Bio-Natto Alternative zu Jogurt Kaffee | Yogurt alternative based on soy, with coffee and natto (Switzerland) | Soyana | Yogurt alternative 73% (water, soybeans, vegan bacterial cultures), natto 16% (soybeans, water, vegan cultures (Bacillus subtilis natto)), coconut blossom sugar, decaffeinated coffee powder 0.6% | 97 | 3.7 | 0.6 | 12 | 10 | 2.5 | 6.9 |  |
| NATUR HARVEST MOON QUARK Alternative | Quark-like alternative based on soy, nature (Austria) | Harvest Moon | Water, coconut milk (coconut, water), coconut oil, starch, faba bean protein, almond protein, sea salt, vegan cultures (S. thermophilus, L. delbrueckii subsp. bulgaricus). | 127 | 10.7 | 9.5 | 4.9 | 0.4 |  | 2.8 |  |
| new roots Blueberry | Yogurt alternative based on mixed substrate, with blueberries | new roots | Cashew–chickpea drink 85% (water, cashew nuts 14%, chickpeas [pasteurized]), blueberries 8.1%, raw cane sugar, lemon juice, stabilizer: pectin, vegan yogurt cultures | 104 | 5.2 | 0.9 | 11 | 7 |  | 2.4 |  |
| new roots Chocolate | Yogurt alternative based on mixed substrate, with chocolate (Switzerland) | new roots | Cashew–chickpea drink 84% (water, cashew nuts 14%, chickpeas [pasteurized]), raw cane sugar, chocolate 1.8% (cocoa mass, cocoa butter, cane sugar, vanilla), cocoa powder 1.3%, stabilizer: pectin, vegan yogurt cultures | 103 | 5.4 | 1 | 11 | 6.7 |  | 2.5 |  |
| new roots Coffee | Yogurt alternative based on mixed substrate, with coffee (Switzerland) | new roots | Cashew–chickpea drink 85% (water, cashew nuts 14%, chickpeas [pasteurized]), raw cane sugar, coffee powder 0.8%, stabilizer: pectin, vegan yogurt cultures | 112 | 5.5 | 1 | 13 | 7.5 |  | 2.6 |  |
| new roots Himbeere | Yogurt alternative based on mixed substrate, with raspberries (Switzerland) | new roots | Cashew–chickpea drink 85% (water, cashew nuts 14%, chickpeas [pasteurized]), raspberries 6.7%, raw cane sugar, lemon juice, stabilizer: pectin, vegan yogurt cultures | 104 | 5.4 | 0.9 | 11 | 7.1 |  | 2.5 |  |
| new roots Nature | Yogurt alternative based on mixed substrate, nature (Switzerland) | new roots | Cashew–chickpea drink 99% (water, cashew nuts 14%, chickpeas [pasteurized]), stabilizer: pectin, vegan yogurt cultures | 89 | 6.4 | 1.1 | 4.6 | 0 |  | 2.8 |  |
| new roots Stracciatella | Yogurt alternative based on mixed substrate, with stracciatella (Switzerland) | new roots | Cashew–chickpea drink 82% (water, cashew nuts 14%, chickpeas [pasteurized]), raw cane sugar, chocolate chips 2.3% (cocoa mass, cocoa butter, cane sugar), vanilla extract, lemon juice, stabilizer: pectin, vegan yogurt cultures | 130 | 6.5 | 1.7 | 15 | 10 |  | 2.4 |  |
| new roots Vanille | Yogurt alternative based on mixed substrate, with vanille (Switzerland) | new roots | Cashew–chickpea drink 88% (water, cashew nuts 14%, chickpeas [pasteurized]), raw cane sugar, vanilla extract 0.04%, vanilla seeds 0.02%, lemon juice, stabilizer: pectin, vegan yogurt cultures | 113 | 5.7 | 1 | 12 | 7.8 |  | 2.5 |  |
| Quark Alternative | Quark-like alternative based on soy, nature (France) | Alnatura | Soy drink 99% (water, dehulled soybeans 13%), thickener: pectin, lactic acid bacteria cultures | 53 | 3.3 | 0.5 | 0.25 | 0.25 | 0.25 | 5.7 |  |
| Sojade À LA GRECQUE NATURE | Greek style yogurt-like alternative based on soy, nature (France) | Sojade | Soya juice 99% (water, soya beans 11.9%), thickener: pectin, selected live cultures | 53 | 3.3 | 0.5 | 1.4 | 0 |  | 5.7 |  |
| Sojade ALTERNATIVE ZU QUARK NATUR | Quark-like alternative based on soy, nature (France) | Sojade | Soy preparation 99% (water, soybeans 11.9%), thickener: pectin, selected cultures | 53 | 3.3 | 0.5 | 0 | 0 |  | 5.7 |  |
| Sojade APRICOT | Yogurt alternative based on soy, with apricot (France) | Sojade | Soy preparation 78% (water, soybeans 10.8%), apricot 10%, raw cane sugar, thickeners: tapioca starch and agar-agar, natural apricot flavoring, selected cultures | 82 | 2.1 | 0.3 | 11.8 | 11.4 | 0.4 | 3.8 |  |
| Sojade BLUEBERRY | Yogurt alternative based on soy, with blueberries (France) | Sojade | Soy preparation 78% (water, soybeans 10.8%), blueberries 10.5%, raw cane sugar, natural blueberry flavoring, thickeners: tapioca starch and locust bean gum, selected cultures | 78 | 2.2 | 0.3 | 10.5 | 9.6 |  | 3.7 |  |
| Sojade FRAISE | Yogurt alternative based on soy, with strawberries (France) | Sojade | Soya juice 78% (water, soya beans 10.8%), strawberry 9%, raw cane sugar, thickeners: tapioca starch, agar-agar, natural strawberry flavoring, concentrated lemon juice, selected live cultures including Bifidobacterium and Lactobacillus acidophilus | 75 | 2.2 | 0.3 | 9.8 | 9.5 |  | 3.8 |  |
| Sojade MANGUE SANS SUCRE AJOUTÉ | Yogurt alternative based on soy, with mango (France) | Sojade | Soy preparation 80% (water, soybeans 10.8%), mango 12.4%, apple juice concentrate, tapioca starch, natural mango flavoring, selected cultures including Bifidobacterium and Lactobacillus acidophilus | 65 | 2.2 | 0.3 | 7.2 | 6.1 |  | 3.9 |  |
| Sojade MYRTILLE CERISE SANS SUCRE AJOUTÉ | Yogurt alternative based on soy, with cherries (France) | Sojade | Soy preparation 81% (water, soybeans 10.8%), fruits 11% (blueberries 64%, cherries 36%), apple juice concentrate, tapioca starch, natural blueberry flavoring, natural cherry flavoring, selected cultures including Bifidobacterium and Lactobacillus acidophilus | 59 | 2.3 | 0.3 | 5.6 | 4.8 | 0.5 | 3.9 |  |
| Sojade NATURAL | Yogurt alternative based on soy, nature (France) | Sojade | Soy preparation 99% (water, soybeans 10.8%), selected cultures including Bifidobacterium and Lactobacillus acidophilus | 46 | 2.9 | 0.4 | 0 | 0 | 0.1 | 4.8 |  |
| Sojade RASPBERRY PASSION FRUIT | Yogurt alternative based on soy, with raspberries and passion fruit (France) | Sojade | Soy preparation 78% (water, soybeans 10.8%), raw cane sugar, raspberries 7%, passion fruit juice 2%, tapioca starch, natural passion fruit flavoring, natural raspberry flavoring, thickener: locust bean gum, selected cultures | 78 | 2.1 | 0.3 | 11 | 10.4 |  | 3.8 |  |
| Sojade SKYR ALTERNATIVE NATURAL | Skyr-like alternative based on soy, nature (France) | Sojade | Soy preparation 98.5% (water, soybeans 11%), faba bean protein 1.5%, selected cultures | 64 | 3.6 | 0.5 | 1.4 | 0 |  | 7 |  |
| Sojade SKYR ALTERNATIVE RASPBERRY | Skyr-like alternative based on soy, with raspberries (France) | Sojade | Soya juice 79% (water, soya beans 11%), raspberries 11.8%, raw cane sugar, broad bean protein 1.2%, tapioca starch, locust bean gum, selected live cultures | 84 | 3 | 0.4 | 9 | 7.4 |  | 5.6 |  |
| Sojade VANILLE | Yogurt alternative based on soy, with vanilla (France) | Sojade | Soy preparation 78% (water, soybeans 10.8%), raw cane sugar, maize starch, natural vanilla flavoring, thickener: guar gum, lemon juice concentrate, ground vanilla pods, selected cultures | 83 | 2.1 | 0.3 | 12.2 | 11.6 | 0.2 | 3.8 |  |
| Sojade WITH KEFIR CULTURES BLUEBERRY CHERRY | Kefir-like alternative based on soy, with blueberries and cherries (France) | Sojade | Soya juice 82% (water, soya beans 7.8%), fruits 9% (blueberries 67%, cherries 33%), apple juice concentrate, tapioca starch, natural blueberry flavoring, natural cherry flavoring, live vegan kefir cultures | 34 | 1.5 | 0.2 | 2.2 | 1.9 |  | 2.8 |  |
| Sojade WITH KEFIR CULTURES NATURAL | Kefir-like alternative based on soy, nature (France) | Sojade | Soy preparation 97.4% (water, soybeans 7.8%), apple juice concentrate, kefir cultures | 34 | 1.5 | 0.2 | 2.2 | 1.9 |  | 2.8 |  |
| SOYANANDA bio Heidelbeere Vegane Alternative zu BioFrüchte-Rahmquark | Quark-like alternative based on soy, with blueberries (Switzerland) | Soyana | Tofu 68% (water, soybeans, lactic acid bacteria cultures), blueberries 12.6%, apple syrup, vegetable oils 7.4% (coconut oil, cold-pressed linseed oil), lemon juice concentrate | 187 | 11 | 5 | 13.6 | 11.3 | 1.5 | 7.8 |  |
| SOYANANDA bio Himbeer Vegane Alternative zu BioFrüchte-Rahmquark | Quark-like alternative based on soy, with raspberries (Switzerland) | Soyana | Tofu 68% (water, soybeans, lactic acid bacteria cultures), raspberries 12.6%, apple syrup, vegetable oils 7.4% (coconut oil, cold-pressed linseed oil), lemon juice concentrate | 185 | 11 | 5 | 12.7 | 10 | 2 | 8 |  |
| SOYANANDA bio Mango Vegane Alternative zu BioFrüchte-Rahmquark | Quark-like alternative based on soy, with mango (Switzerland) | Soyana | Tofu 68% (water, soybeans from the EU, lactic acid bacteria cultures), mango 14.5%, apple syrup, vegetable oils 7.4% (coconut oil, cold-pressed linseed oil), lemon juice concentrate, turmeric | 180 | 11 | 5 | 12 | 10 | 1.4 | 7.8 |  |
| SOYANANDA bio Vegane Alternative zu BioSpeisequark nature | Quark-like alternative based on soy, nature (Switzerland) | Soyana | Tofu 100% (water, soybeans, lactic acid bacteria cultures) | 104 | 4.4 | 0.7 | 5.9 | 2.6 | 2.2 | 10.2 |  |
| SOYANANDA Nature bio Fermentierte BioSoya-Alternative zu Jogurt | Yogurt alternative based on soy, nature (Switzerland) | Soyana | Water, 22% soybeans, thickener: agar-agar, selected bacterial cultures | 50 | 2.5 | 0.4 | 2.5 | 1.3 |  | 4.6 |  |
| THE VEGAN COW HAFER JOGU KAFFEE | Yogurt alternative based on oat, with coffee (Germany) | The Vegan Cow | Oat preparation 85% (water, wholegrain oats 10%, coconut fat, waxy maize starch, thickener: locust bean gum, sea salt, yogurt cultures), coffee preparation 15% (water, beet sugar, coffee concentrate, maize starch, thickener: locust bean gum, caramel, coffee extract) | 105 | 4 | 3.2 | 16 | 11 |  | 0.7 |  |
| THE VEGAN COW HAFER JOGU MANGO MARACUJA | Yogurt alternative based on oat, with mango and maracuja (Germany) | The Vegan Cow | Oat preparation 85% (water, wholegrain oats 10%, coconut fat, waxy maize starch, thickener: locust bean gum, sea salt, yogurt cultures), mango–passion fruit preparation 15% (mango purée, beet sugar, passion fruit juice concentrate, water, maize starch) | 102 | 4.5 | 3.7 | 15 | 9.4 |  | 0.6 |  |
| THE VEGAN COW HAFER JOGU NATUR | Yogurt alternative based on oat, nature (Germany) | The Vegan Cow | Oat preparation 91% (water, wholegrain oats 11%, yogurt cultures), coconut fat, waxy maize starch, thickener: locust bean gum, sea salt | 83 | 4.5 | 3.7 | 9.8 | 5.8 |  | 0.7 |  |
| THE VEGAN COW HAFER JOGU VANILLE | Yogurt alternative based on oat, with vanilla (Germany) | The Vegan Cow | Oat preparation 88% (water, wholegrain oats 10%, coconut fat, waxy maize starch, thickener: locust bean gum, sea salt, yogurt cultures), vanilla preparation 12% (beet sugar, water, maize starch, ground Bourbon vanilla pods, lemon juice concentrate, thickener: locust bean gum) | 108 | 4.1 | 3.3 | 14 | 10 |  | 0.6 |  |
| THE VEGAN COW HAFER JOGU WALDFRUCHT | Yogurt alternative based on oat, with berries (Germany) | The Vegan Cow | Oat preparation 85% (water, wholegrain oats 10%, coconut fat, waxy maize starch, thickener: locust bean gum, sea salt, yogurt cultures), berry preparation 15% (blackberries, beet sugar, water, raspberries, blueberries, elderberry juice concentrate, maize starch, thickener: locust bean gum) | 103 | 4.1 | 3.3 | 16 | 10 |  | 0.7 |  |
| V-LOVE PLANT BASED BIO BLUEBERRY COCONUT | Yogurt alternative based on coconut, with blueberries (Switzerland) | V Love | Water, coconut extract 21%, blueberries 8%, raw cane sugar, waxy maize starch, tapioca starch, natural blueberry flavoring, lemon juice concentrate, thickener: locust bean gum, yogurt cultures, salt | 104 | 7.3 | 6.8 | 8.6 | 4.9 | 0.5 | 0.8 |  |
| V-LOVE PLANT BASED BIO CLASSIC COCONUT | Yogurt alternative based on coconut, nature (Switzerland) | V Love | Water, coconut extract 29%, starches (tapioca, potato, waxy maize), thickener: locust bean gum, Streptococcus thermophilus, Lactobacillus bulgaricus, salt | 105 | 10 | 9.5 | 2.7 | 0.7 | 0.25 | 1 |  |
| V-LOVE PLANT BASED BIO MANGO-PASSION COCONUT | Yogurt alternative based on coconut, with mango and passion fruit (Switzerland) | V Love | Water, coconut extract 23%, mango purée 7%, sugar, passion fruit juice concentrate 3.5%, starches (tapioca, potato, waxy maize), thickener: locust bean gum, Streptococcus thermophilus, Lactobacillus bulgaricus, salt, flavoring, lemon juice concentrate, turmeric extract | 109 | 8 | 7.6 | 8.2 | 5.3 | 0.25 | 0.9 |  |
| V-LOVE PLANT BASED CHOCHOLATE ALMOND | Yogurt alternative based on almond, with chocolate (Switzerland) | V Love | Water, almond purée 10%, raw cane sugar, starches, low-fat cocoa powder 2%, cocoa butter, lemon juice concentrate, fermentation cultures | 102 | 5.6 | 0.6 | 10 | 7.2 | 0.7 | 2.6 |  |
| V-LOVE PLANT BASED HIMBEERE | Yogurt alternative based on almond, with raspberries (Switzerland) | V Love | Water, almond purée 9.4%, raspberry pieces 6.4%, sugar, tapioca starch, raspberry purée from concentrate 1.6%, modified waxy maize starch, natural flavoring, lemon juice concentrate, black carrot juice concentrate, lactic acid bacteria | 92 | 5 | 0.6 | 9 | 6 | 0.25 | 2.6 |  |
| V-LOVE PLANT BASED MANDEL MOKKA | Yogurt alternative based on almond, with almond and coffee (Switzerland) | V Love | Water, almond purée 9.9%, sugar, tapioca starch, coffee extract 0.7%, modified maize starch, natural flavoring, lactic acid bacteria | 94 | 5 | 0.25 | 10 | 7 | 0 | 2.3 |  |
| V-LOVE PLANT BASED SOJA BIRCHERMÜSLI | Yogurt alternative based on soy, Birchermuesli-style (Switzerland) | V Love | Water, fruits 10% (apples 46%, banana purée 21%, peaches 14%, pears 11%, strawberries 8%), dehulled soybeans 6%, raw cane sugar, cereal flakes 1.5% (wheat flakes 88%, oat flakes 12%), tapioca starch, yogurt cultures, lemon juice concentrate | 78 | 2.1 | 0.3 | 11 | 7.8 | 0.9 | 3.3 |  |
| V-LOVE PLANT BASED SOJA CLASSIC | Yogurt alternative based on soy, nature (Switzerland) | V Love | Water, dehulled soybeans 8%, tapioca starch, bacterial cultures: Streptococcus thermophilus, Lactobacillus bulgaricus, Bifidobacterium lactis, Lactobacillus acidophilus | 46 | 2.5 | 0.4 | 1.8 | 0 | 0.6 | 3.7 |  |
| V-LOVE PLANT BASED SOJA MANGO | Yogurt alternative based on soy, with mango (Switzerland) | V Love | Water, raw cane sugar, mango purée 7%, dehulled soybeans 6%, mango 5%, tapioca starch, lemon juice concentrate, yogurt cultures | 76 | 2 | 0.3 | 11 | 9.4 | 0.7 | 3.1 |  |
| V-LOVE PLANT BASED SOJA STRAWBERRY | Yogurt alternative based on soy, with strawberries (Switzerland) | V Love | Water, strawberries 11%, raw cane sugar, dehulled soybeans 6%, tapioca starch, lemon juice concentrate, yogurt cultures | 76 | 2 | 0.3 | 11 | 9.5 | 0.7 | 3.1 |  |
| VEMONDO BLUEBERRY SOYA Sojacrème | Yogurt alternative based on soy, with blueberries (Germany) | Vemondo | Water, soybeans 11%, sugar, blueberries 5.5%, modified starch, blueberry juice from concentrate 1.1%, acidity regulators (citric acid, sodium citrates, calcium citrates, malic acid), coloring carrot concentrate, starch, natural flavoring, sea salt, thickener: locust bean gum, apple extract, yogurt cultures | 72 | 1.8 | 0.2 | 9.9 | 8 | 1 | 3.6 |  |
| VEMONDO CLASSIC SOYA Sojacrème | Yogurt alternative based on soy, nature (Germany) | Vemondo | Water, 13.3% soybeans, modified starch, starch, sea salt, natural flavoring, yogurt cultures | 46 | 2.2 | 0.3 | 2.1 | 0.6 | 0.7 | 4.2 |  |
| VEMONDO PFIRSICH KOKOSCREME AUF BASIS VON KOKOS | Yogurt alternative based on coconut, with peach (Poland) | Vemondo | Coconut extract 70.9% (water, coconut extract), peach 12%, agave syrup, starch, white grape juice concentrate, lemon juice concentrate, natural peach flavoring, lactic acid cultures (S. thermophilus, L. delbrueckii subsp. bulgaricus) | 105 | 5.3 | 5.1 | 13.4 | 8.4 | 0.2 | 0.7 |  |
| VEMONDO VANILLE SOYA Sojacrème | Yogurt alternative based on soy, with vanilla (Germany) | Vemondo | Water, soybeans 11%, sugar, modified starch, acidity regulators (citric acid, sodium citrates, calcium citrates), starch, natural flavoring, coloring carrot concentrate, sea salt, thickener: locust bean gum, ground extracted vanilla pods, yogurt cultures | 69 | 1.8 | 0.2 | 9.3 | 7.5 | 0.7 | 3.5 |  |
| VEMONDO VEGURT CHOCOLAT | Yogurt alternative based on mixed substrate, with chocolate (Switzerland) | Vemondo | 85% cashew–chickpea drink (water, 14% cashew nuts, chickpeas), raw cane sugar, chocolate 1.8% (cocoa mass, cocoa butter, cane sugar, vanilla), cocoa powder 1.3%, stabilizer: pectins, yogurt cultures | 103 | 5.4 | 1 | 11 | 6.7 | 0 | 2.5 |  |
| VEMONDO VEGURT HIMBEERE | Yogurt alternative based on mixed substrate, with raspberries (Switzerland) | Vemondo | 85% cashew–chickpea drink (water, 14% cashew nuts, chickpeas), raspberries 6.7%, raw cane sugar, lemon juice, stabilizer: pectins, yogurt cultures | 103 | 5.4 | 0.9 | 11 | 7.1 | 0 | 2.5 |  |
| VEMONDO VEGURT MOCCA | Yogurt alternative based on mixed substrate, with coffee (Switzerland) | Vemondo | 85% cashew–chickpea drink (water, 14% cashew nuts, chickpeas), raw cane sugar, coffee powder 0.8%, stabilizer: pectins, yogurt cultures | 112 | 5.5 | 1 | 13 | 7.5 | 0 | 2.6 |  |
| VEMONDO VEGURT NATURE | Yogurt alternative based on mixed substrate, nature (Switzerland) | Vemondo | 99% cashew–chickpea drink (water, 14% cashew nuts, chickpeas), stabilizer: pectins, yogurt cultures | 89 | 6.4 | 1.1 | 4.6 | 0 | 1 | 2.8 |  |

**Macronutrient profiles of fermented plant-based yogurt and yogurt-like alternatives**

**Supplementary table S2.** Macronutrient profiles of plain fermented plant-based yogurt alternatives across different plant substrates compared with dairy yogurt (reference).

| **Substrate** | **Sample size** | **Energy (kcal/100g)**  Median (Range) | **Carbohydrates (g/100g)**  Median (Range) | **Total sugars (g/100g)**  Median (Range) | **Fat(g/100g)**  Median (Range) | **Saturated Fat (g/100g)**  Median (Range) | **Protein**  **(g/100g)**  Median (Range) | **Salt**  **(g/100g)**  Median (Range) |
| --- | --- | --- | --- | --- | --- | --- | --- | --- |
| **Dairy yogurt** |  | 66 | 4.5 | 4.5 | 3.6 | 2.2 | 3.9 | 0.1 |
| **Soy-based** | n=8 | 46.00*  (42.00-50.00) | 1.80*  (0.00-2.50) | 0.00*  (0.00-1.30) | 2.50*  (2.20-2.90) | 0.40**  (0.30-0.40) | 4.20  (3.70-4.80) | 0.10  (0.00-0.24) |
| **Coconut-based** | n=7 | 95.00*  (77.00-105.00) | 5.00  (2.70-7.70) | 0.60*  (0.00-1.00) | 7.9*  (4.8-10) | 7.50*  (4.60-9.50) | 0.80*  (0.50-1.00) | 0.05  (0.00-0.18) |
| **Mixed substrate-based** | n=3 | 89.00 (57.00-89.00) | 4.60  (2.10-4.60) | 0.00  (0.00-0.80) | 6.4  (3.5-6.4) | 1.10  (1.10-2.00) | 2.80  (2.80-4.00) | 0.00 (  0.00-0.40) |
| **Oat-based** | n=3 | 83.00  (77.00-95.00) | 11.00  (9.80-11.40) | 3.20  (2.80-5.80) | 4.5  (3.5-5.4) | 3.70  (2.70-4.60) | 0.25  (0.20-0.70) | 0.09  (0.05-0.10) |
| **Almond-based** | n=3 | 53.00  (52.00-55.00) | 4.40  (2.80-5.00) | 0.25  (0.25-0.80) | 3.40  (3.30-6.70) | 0.40  (0.30-0.50) | 1.60  (0.25-2.50) | 0.08  (0.01-0.10) |
| **Mixed substrate-based** | n=3 | 89.00 (57.00-89.00) | 4.60  (2.10-4.60) | 0.00  (0.00-0.80) | 6.4  (3.5-6.4) | 1.10  (1.10-2.00) | 2.80  (2.80-4.00) | 0.00 (  0.00-0.40) |

*, **, *** denote p < 0.05, p < 0.01, p < 0.001. p-values were derived from one-sample Wilcoxon Signed-Rank Tests comparing macronutrient contents of dairy yogurt with soy- and coconut-based alternatives only, due to limited sample sizes in other substrate categories. Macronutrient values for dairy yogurt were obtained from the Swiss Food Composition Database (18)

**Supplementary table S3.** Macronutrient profiles of fermented plant-based yogurt-like alternatives across different plant substrates compared with dairy yogurt (reference).

| **Substrate** | **Sample size** | **Energy (kcal/100g)** | **Fat**  **(g/100g)** | **Saturated Fat (g/100g)** | **Carbohydrates (g/100g)** | **Total sugars (g/100g)** | **Protein**  **(g/100g)** | **Salt**  **(g/100g)** |
| --- | --- | --- | --- | --- | --- | --- | --- | --- |
|  |  | Median (Range) | Median (Range) | Median (Range) | Median (Range) | Median (Range) | Median (Range) | Median (Range) |
| **Dairy yogurt** |  | 66 | 3.6 | 2.2 | 4.5 | 4.5 | 3.9 | 0.1 |
| **Yogurt-like alternatives**  **(Plain)** | n=8 | 57.00 (34.00-127.00) | 3.30  (1.50-10.70) | 0.50  (0.20-9.50) | 1.40*  (0.00-5.90) | 0.12*  (0.00-2.60) | 5.70*  (2.80-10.20) | 0.11  (0.09-0.33) |
| **Yogurt-like alternatives**  **(Flavoured)** | n=8 | 83.00 (34.00-187.00) | 3.00  (1.50-11.00) | 0.45  (0.20-5.00) | 8.10*  (2.20-13.60) | 7.20  (1.90-11.30) | 7.5  (2.8-8.00) | 0.12  (0.00-0.24) |

*, **, *** denote p < 0.05, p < 0.01, p < 0.001. p-values were derived from one-sample Wilcoxon Signed-Rank Tests comparing macronutrient contents of dairy yogurt with each plant-based substrate. Macronutrient values for dairy yogurt were obtained from the Swiss Food Composition Database (18)

**Supplementary table S4.** Macronutrient profiles of flavored fermented plant-based yogurt alternatives across different plant substrates compared with dairy yogurt (reference).

| **Substrate** | **Sample size** | **Energy (kcal/100g)** | **Fat**  **(g/100g)** | **Saturated Fat (g/100g)** | **Carbohydrates (g/100g)** | **Total sugars (g/100g)** | **Protein**  **(g/100g)** | **Salt**  **(g/100g)** |
| --- | --- | --- | --- | --- | --- | --- | --- | --- |
|  |  | Median (Range) | Median (Range) | Median (Range) | Median (Range) | Median (Range) | Median (Range) | Median (Range) |
| **Dairy yogurt** |  | 66 | 3.6 | 2.2 | 4.5 | 4.5 | 3.9 | 0.1 |
| **Soy-based yogurt alternatives** | n=22 | 73.00**  (58.00-97.00) | 2.10***  (1.80-3.70) | 0.30***  (0.20-0.90) | 9.85***  (4.80-13.00) | 8.10***  (4.40-11.60) | 3.65**  (3.10-6.90) | 0.10  (0.00-0.23) |
| **Coconut-based yogurt alternatives** | n=15 | 109.00***  (95.00-138.00) | 7.20***  (5.30-8.00) | 6.60***  (5.10-7.70) | 11.80***  (8.20-15.50) | 7.70***  (4.40-11.00) | 0.70***  (0.60-1.70) | 0.04***  (0.02-0.07) |
| **Oat-based yogurt alternatives** | n=6 | 102.50*  (88.00-108.00) | 4.05  (2.90-4.50) | 3.25*  (2.50-3.70) | 15.15*  (14.00-16.00) | 9.70*  (8.50-11.00) | 0.60*  (0.20-0.70) | 0.10  (0.09-0.12) |
| **Almond-based yogurt alternatives** | n=3 | 94.00  (92.00-102.00) | 5.00  (5.00-5.60) | 0.60  (0.25-0.60) | 10.00  (9.00-10.00) | 7.00  (6.00-7.20) | 2.60  (2.30-2.60) | 0.05  (0.00-0.05) |
| **Mixed substrate-based yogurt alternatives** | n=12 | 104.50**  (82.00-130.00) | 5.40**  (2.90-6.50) | 1.00**  (0.90-2.50) | 11.00**  (9.90-15.00) | 7.50**  (6.70-12.00) | 2.50**  (2.40-4.00) | 0.01**  (0.00-0.04) |

*, **, *** denote p < 0.05, p < 0.01, p < 0.001. p-values were derived from one-sample Wilcoxon Signed-Rank Tests comparing macronutrient contents of dairy yogurt with each plant-based substrate (except for almond-based yogurt alternatives, due to limited sample size). Macronutrient values for dairy yogurt were obtained from the Swiss Food Composition Database (18)

**Fiber content of fermented plant-based yogurt and yogurt-like alternatives**

**Supplementary table S5.** Fiber content of plain, flavored fermented plant-based yogurt alternatives and yogurt-like alternatives

| **Substrate** | **Sample size** | **Fiber (g/100g)** |
| --- | --- | --- |
|  |  | **Median (Range)** |
| Dairy yogurt |  | 0 |
| **Plain plant-based yogurt alternatives** | | |
| Soy-based yogurt alternatives | n=6 | 0.6 (0.1 - 0.9) |
| Coconut-based yogurt alternatives | n=3 | 0.25 (0-0.5) |
| Oat-based yogurt alternatives | n=0 | n.d. |
| Almond-based yogurt alternatives | n=2 | 1.6 (1.3 - 1.9) |
| Mixed substrate-based yogurt alternatives | n=1 | 1 |
| **Flavored** **plant-based yogurt alternatives** | | |
| Soy-based yogurt alternatives | n=15 | 0.9 (0.2 - 2.5) |
| Coconut-based yogurt alternatives | n=5 | 0.2 (0-0.5) |
| Oat-based yogurt alternatives | n=0 | n.d. |
| Almond-based yogurt alternatives | n=3 | 0.25 (0-0.7) |
| Mixed substrate-based yogurt alternatives | n=3 | 0 |
| **Fermented plant-based yogurt-like alternatives** | | |
| Plain yogurt-like alternatives | n=3 | 1.3 (0.25 - 2.2) |
| Flavoured yogurt-like alternatives | n=6 | 1.25 (1-2) |

Macronutrient values for dairy yogurt were obtained from the Swiss Food Composition Database (18)

**Micronutrient modelling based on the recommended daily portion size**

**Supplementary table S6.** Modelled daily micronutrient contribution from a recommended portion of 175 g of plain yogurt (20), comparing plain dairy yogurt with substitutions using plain soy- and coconut-based yogurt alternatives. For each micronutrient, absolute intake per portion and the corresponding percentage of the adult recommended dietary allowance (RDA) are presented, minimum and maximum ranges were reported to reflect sex-specific intake requirements (20).

| **Micronutrient** | **Unit** | **Dairy yogurt, plain, full-fat**  (Micronutrient intake in daily recommended portion) | % of RDA (adults) | **Soy yogurt alternative, plain**  (Micronutrient intake in daily recommended portion) | % of RDA (adults) | **Coconut yogurt alternative, plain**  (Micronutrient intake in daily recommended portion) | % of RDA (adults) |
| --- | --- | --- | --- | --- | --- | --- | --- |
| Vitamin A activity, RE | µg | 68.25 | 9.10-10.50 | 1.75 | 0.23-0.27 | 1.75 | 0.23-0.27 |
| Vitamin B_1_ (Thiamine) | mg | 0.04 | 4.17 | 0.02 | 2.08 | 0.00 | ≤ 1.00 |
| Vitamin B₂ (Riboflavin) | mg | 0.32 | 19.69 | 0.01 | ≤ 1.00 | 0.00 | ≤ 1.00 |
| Vitamin B_3_ (Niacin) | mg | 0.16 | 1.21 | 0.09 | 0.67 | 0.18 | 1.35 |
| Vitamin B_5_ (Pantothenic acid) | mg | 0.58 | 11.55 | 0.07 | 1.40 | 0.05 | 1.05 |
| Vitamin B_6_ (Pyridoxine) | mg | 0.07 | 4.12-4.38 | 0.01 | ≤ 1.00 | 0.00 | ≤ 1.00 |
| Vitamin B_9_ (Folate) | µg | 8.75 | 2.65 | 32.55 | 9.86 | 0.88 | 0.27 |
| Vitamin B_12_ (Cobalamin) | µg | 0.53 | 13.13 | 0.00 | ≤ 1.00 | 0.00 | ≤ 1.00 |
| Vitamin C (Ascorbic acid) | mg | 3.50 | 3.18-3.68 | 0.44 | ≤ 1.00 | 0.00 | ≤ 1.00 |
| Vitamin D (Calciferol) | µg | 0.18 | 1.17 | 0.26 | ≤ 1.00 | 0.00 | ≤ 1.00 |
| Vitamin E (α-tocopherol) | mg | 0.18 | 1.35-1.59 | 0.40 | 3.10-3.66 | 0.00 | ≤ 1.00 |
| Potassium (K) | mg | 297.50 | 8.50 | 192.50 | 5.50 | 161.00 | 4.60 |
| Sodium (Na) | mg | 85.75 | 4.29 | 59.50 | 2.98 | 43.75 | 2.19 |
| Chloride (Cl) | mg | 210.00 | 6.77 | 42.00 | 1.35 | 119.00 | 3.84 |
| Calcium (Ca) | mg | 245.00 | 24.50-25.79 | 24.50 | 2.45-2.58 | 8.75 | 0.88-0.92 |
| Magnesium (Mg) | mg | 21.00 | 6.00-7.00 | 29.75 | 8.50-9.92 | 16.98 | 4.85-5.66 |
| Phosphorus (P) | mg | 192.50 | 35.00 | 84.00 | 15.27 | 38.50 | 7.00 |
| Iron (Fe) | mg | 0.00 | ≤ 1.00 | 0.70 | 4.38-6.36 | 0.26 | 1.64-2.39 |
| Iodine (I) | µg | 28.00 | 18.67 | n.d. | n.d. | n.d. | n.d. |
| Zinc (Zn) | mg | 0.70 | 4.29-9.33 | 0.53 | 3.22-7.00 | 0.18 | 1.07-2.33 |

Median micronutrient values were obtained from the Swiss Food Composition Database (18)
